# Applicability of a “Multi-Stage Pulse Labeling” ^15^N Approach to Phenotype N Dynamics in Maize Plant Components during the Growing Season

**DOI:** 10.3389/fpls.2017.01360

**Published:** 2017-08-04

**Authors:** Amanda de Oliveira Silva, James J. Camberato, Tristan Coram, Timothy Filley, Tony J. Vyn

**Affiliations:** ^1^Department of Agronomy, Kansas State University Manhattan, KS, United States; ^2^Agronomy Department, Purdue University West Lafayette, IN, United States; ^3^Australian Grain Technologies Adelaide, SA, Australia

**Keywords:** isotopic N, maize hybrids, plant N uptake, plant component N distribution, N recovery efficiency

## Abstract

**Highlights**

- This work utilizes “multi-stage pulse labeling” ^15^N applications, primarily during reproductive growth stages, as a phenotyping strategy to identify maize hybrids with superior N use efficiency (NUE) under low N conditions.

Research using labeled isotopic N (^15^N) can precisely quantify fertilizer nitrogen (N) uptake and organ-specific N allocation in field crops such as maize (*Zea mays* L.). The overall research objective was to study plant N uptake patterns potentially correlated with N use efficiency (NUE) in field-grown maize hybrids using a “multi-stage pulse labeling” ^15^N phenotyping strategy with an emphasis on the reproductive period. Five hybrids varying in NUE were compared under zero N fertilizer application (0N) plus a moderate rate of 112 kg N ha^−1^ (112N) in 2013 (2 locations) and 2014 growing seasons. The equivalent of 3.2 (2013) to 2.1 (2014) kg of ^15^N ha^−1^, as labeled Ca(^15^NO_3_)_2_, was injected into soil on both sides of consecutive plants at multiple stages between V14 and R5. Aboveground plant biomass was primarily collected in short-term intervals (4–6 days after each ^15^N application) in both years, and following a single long-term interval (at R6 after ^15^N injection at R1) in 2014. Averaged across hybrids and site-years, the moderate N rate (112N) increased absolute ^15^N uptake at all stages; however, plants in the 0N treatment allocated proportionally more ^15^N to reproductive organs. Before flowering, short-term recovery of ^15^N (^15^Nrec) totaled ~0.30 or 0.40 kg kg^−1^ of the ^15^N applied, and ~50% of that accumulated ^15^Nu was found in leaves and 40% in stems. After flowering, plant ^15^Nrec totaled ~0.30 kg kg^−1^ of ^15^N applied, and an average 30% of accumulated ^15^Nu was present in leaves, 17% in stems, and the remainder—usually the majority—in ears. At the R5 stage, despite a declining overall rate of ^15^N uptake per GDD thermal unit, plant ^15^Nrec represented ~0.25 kg kg^−1^ of ^15^N applied, of which ~65% was allocated to kernels. Overall long-term ^15^Nrec during grain filling was ~0.45 and 0.70 kg kg^−1^ of total ^15^N applied at R1 with 0 and 112N, respectively, and most (~77%) ^15^N uptake was found in kernels. The “multi-stage pulse labeling” technique proved to be a robust phenotyping strategy to differentiate reproductive-stage N uptake/allocation patterns to plant organs and maize efficiencies with newly available fertilizer N.

## Introduction

Maize is one of the most important crops cultivated for food and feed production worldwide and it is also important for biofuel production in some countries. Maize requires significant amounts of N to maximize yield (Coelho et al., 1991; Fageria and Baligar, 2005). Application of N fertilizer increased over the years from 1960 to 1980 (Cassman et al., 2002; Tilman et al., 2002; Van Cleemput et al., 2008; Scharf, 2015), but since then overall N rates utilized for maize production in the United States have stabilized (USDA-ERS, 2013). However, maize may still only take up about 50% of total N fertilizer applied (Dobermann and Cassman, 2004; Silva et al., 2006; Van Cleemput et al., 2008; Ciampitti and Vyn, 2014). Increasing the efficiency by which plants use available N is crucial to optimize crop yield potential, reduce the costs of N inputs, and subsequently avoid N losses to the environment (Raun and Johnson, 1999; Stevens et al., 2005).

The quantitative nature of genes controlling N use efficiency (NUE) integrated with the complex N cycles in soil-plant systems and in alternate crop management and environmental scenarios make it challenging to improve NUE (Amado et al., 2002; Mosier et al., 2004; Fageria and Baligar, 2005; Ladha et al., 2005; Mi et al., 2005; Coque et al., 2008). Previous improvements in maize NUE in field research programs have largely been based on grain yield and N uptake evaluations of diverse genetic materials advanced from both traditional and transgenic breeding approaches (Guo et al., 2014) with or without integrated agronomic practices (often involving multiple N rates) in various environmental conditions (Duvick, 1984; Ciampitti and Vyn, 2011). Because NUE is directly related to yield improvement, NUE gains occur when maize plants take up more N from soil and fertilizer N sources [preferably with a high N recovery efficiency (NRE)] and, thereafter, produce maximum grain yield per unit of plant N uptake (known as N internal efficiency; NIE) (Moll et al., 1982; Cassman et al., 2003; Dobermann, 2005; Mi et al., 2005; Coque and Gallais, 2007; Ciampitti and Vyn, 2012).

Raun and Johnson (1999) stated that low NUE (≈33 kg grain kg^−1^) in crop production was a consequence of the excessive use of N fertilizer. Thus, to optimize crop yield and improve NUE it is necessary to develop strategies that synchronize optimal timings of N application with plant N requirements (Cassman et al., 2002; Tilman et al., 2002; Dobermann, 2005; Fageria and Baligar, 2005; Mueller et al., 2014). Regardless of whether NUE is determined by the simple or the more robust difference method, these methods are unable to distinguish the in-season soil vs. fertilizer sources of N uptake, allocation, and remobilization by plants. The use of isotopic N (^15^N) allows closer estimation of the current N uptake by plants. Additionally, multiple ^15^N applications during the growing season can help to precisely determine the fate of the most recent inorganic N uptake and its initial organ-specific allocation inside plants.

Most commonly, ^15^N experiments involving crop response are developed under controlled environments in greenhouses (Pan et al., 1986; Schmidt and Scrimgeour, 2001; Rimski-Korsakov et al., 2009) or in the field by the use of chambers or lysimeters (Portela et al., 2006; O'Brien et al., 2012). In the greenhouse, Friedrich and Schrader (1979), evaluated maize N remobilization patterns during the grain filling period. They applied labeled ^15^N as nutrient solution to maize plants during the entire vegetative period up to the R1 stage and harvested plants from 1 to 7 weeks after R1. More recently, Paponov and Engels (2005), evaluated plant N partitioning also in a greenhouse study. They applied ^15^N to the soil surface 12 days after silking and harvested maize plants 14 and 47 days after silking.

Research with ^15^N techniques in large-scale field experiments has been limited due to the labeled product costs and elaborate sampling procedure in mass spectrometry analysis, which requires specialized technical assistance. Because of high costs of ^15^N products, researchers using isotopic N in open and/or closed systems usually apply ^15^N jointly with N fertilizer treatments at low enrichment rates (~5% atom ^15^N) or use ^15^N-depleted fertilizers (Coelho et al., 1991; Boaretto et al., 2004; Gallais et al., 2006).

In some cases, fertilizers incorporating labeled N were applied early- to mid-season, so that studies could examine N accumulation, remobilization, and recovery in maize plants at flowering or physiological maturity (Cliquet et al., 1990a; Ma et al., 1998; Stevens et al., 2005; Gallais et al., 2006; Silva et al., 2006; Ning et al., 2017). Several experiments conducted in the field have also used ^15^N with traditional N management practices to analyze the “long-term” N accumulation in the plants within a season (Coelho et al., 1991; Gallais et al., 2006; Duete et al., 2009). Gallais et al. (2006), mainly focused on the evaluation of sources of N accumulated in the grain at physiological maturity. They used two ^15^N application techniques: ^15^N was applied at either the beginning of stem elongation or at R1 and plants were harvested multiple times after these two applications (at R1, R1+ 15, 25, and 35 days, and at R6). They found that up to 55% of grain N originates from post-silking N uptake, while the remaining comes from N remobilization from the vegetative organs. Such “long-term” approaches seems to be more appropriate to quantify the remobilization of N accumulated in vegetative organs to the grain at maturity and the total N accumulation during a certain period than to estimate the fate of newest N uptake within the plant during the reproductive period.

There has only been a single field study in which ^15^N was applied to maize more than once in the growing season with plants harvested shortly after the ^15^N application. In that single-year and single-location experiment, Ta and Weiland (1992), compared two historic hybrids (crosses with a common B73 inbred) with different leaf senescence characteristics at two pre-plant N rates. The authors applied 50 mg of ^15^N to the soil beside single maize plants at three development stages (V14, R1, and R1 + 22 days) and these individual plants were harvested 3 days later (and at 35, 45, and ~67 days after the R1 application). Their study estimated that ~45% of the ^15^N applied was absorbed within 3 days and that more of the labeled N went to the ears with later ^15^N application times, or a longer remobilization period. Biomass and N uptake measurements from single plants, though indicative of trends, are never as precise as those from larger plant samples.

To date, the Ta and Weiland's (1992), study above is the only known report involving ^15^N applied to field-based maize a single time after the R1 stage. The knowledge gap concerning the most recent N uptake during the reproductive period is important because modern maize hybrids respond to N much differently than hybrids of earlier decades. More recent hybrids generally have higher N internal efficiency, and much more of their total N uptake occurs post-flowering (Ciampitti and Vyn, 2012, 2013; Chen et al., 2015; Mueller and Vyn, 2016). The gains in total N uptake with modern hybrids is not because of any higher leaf N concentrations at flowering (they may even be lower: Chen et al., 2015), but mostly because of the enhanced N accumulation during reproductive growth (Chen et al., 2015; Mueller and Vyn, 2016). Just how late in the grain filling period new N is taken up from the soil with more recent hybrids is still unidentified. Precise measurements of the efficiency with which modern maize hybrids uptake and allocate new N in its organs at multiple reproductive stages remains unknown.

Integrating cutting-edge cropping systems with high precision management and more resource-efficient genotypes are fundamental to increase crop production. New phenotyping approaches are necessary to identify NUE differences among maize hybrids during the growing season, and to better understand the when and where questions of hybrids that are supposedly superior in fertilizer N efficiencies.

The present study attempted to validate the utilization of the “multi-stage pulse labeling” ^15^N approach in field-grown maize as an advanced phenotyping technique to identify key mechanisms in N uptake, allocation, and partitioning during the growing season among hybrids with possible NUE variation. In this research, we modify earlier methods of tracer ^15^N utilization to more precisely estimate the fate of recent N uptake, partitioning, and recovery in maize plant components under different levels of N stress throughout the reproductive period. The research objectives were (i) to utilize “multi-stage pulse labeling” ^15^N application as a high precision phenotyping technique in maize hybrids grown at low N rates; (ii) to quantify ^15^N proportional allocation, and ^15^N fertilizer recovery, in plant components just before and at least four times during the reproductive growth period; and (iii) to examine how DM accumulation in discrete growth intervals impacts total plant ^15^N uptake activity throughout the growing season.

## Materials and methods

### Weather description, management practices, and experimental design

A non-irrigated research study was conducted during two growing seasons (2013–2014) in the US Midwestern Corn Belt region. In 2013, field experiments were established at the Purdue University Agronomy Center for Research and Education (ACRE) near West Lafayette—IN (Lat 40.486675° Lon 87.004635°, elevation 216 m) and at Pinney-Purdue Agricultural Center (PPAC) near Wanatah—IN (Lat 41.445113° Lon 86.943464°, elevation 222 m). At ACRE, soil was a Chalmers silty—clay loam (Fine—silty, mixed, superactive, mesic Typic Endoaquolls) and at PPAC soil was Sebewa loam (Fine—loamy over sandy or sandy—skeletal, mixed, superactive, mesic Typic Argiaquolls). In 2014, one experiment was established at ACRE (Lat 40.493593°, Lon 86.493593°, elevation 216 m). Soil type was a Raub—Brenton complex (Fine-silty, mixed, superactive, mesic Aquic Argiudolls).

Maize field trials were established following soybean [*Glycine max* (L.) Merr.] at all three site-years. In 2013, tillage operations prior to planting maize involved fall chisel plow followed by a spring field cultivator. For the 2014 site, fall plus spring strip-tillage on the no-till soybean stubble preceded maize planting (with precision guidance in all operations).

Experiments were planted with a four—row precision planter (Almaco SeedPro 360) with 76 cm row spacing, to achieve a final plant density of ~79,000 plants ha^−1^. In 2013 plot lengths were shorter (6.7 m) than in 2014 (13.7 m) due to seed supply limitations. Plant populations were evaluated in 5.3 m row length sections of all four rows at the V5 growth stage.

Weather data was acquired on a daily basis from planting to harvest (1 April—May to 31 October 2013 and 2014) from automated weather stations operated by the Indiana State Climate Office proximately located to the research sites. The field experiment at ACRE in 2013 was planted on 14 May and harvested 10 October. The total growing season precipitation was 385 mm; maximum and minimum daily average air temperatures were 26.9 and 14.6°C, respectively, for the entire growing season (Table 1). The field experiment at PPAC in 2013 was planted 5 June and harvested 30 October. Total growing season precipitation was 626 mm; maximum and minimum daily average temperatures were 24.7 and 12.1°C, respectively, for the entire growing season (Table 1). The field experiment at ACRE in 2014 was planted on 25 April and harvested 25 September. Total precipitation was 592 mm and maximum and minimum daily temperatures averaged 26.2 and 13.6°C throughout the growing season (Table 1). Weed pressure was minimal at all locations.

**Table 1:** Pertinent phenology and climate information of isotopic N experiments conducted at ACRE and PPAC locations in 2013 and at ACRE in 2014.

**Location**	**Growth stages**	Plant components harvested^*^						**Biomass harvest date**	**GDD°C (^15^N days)**	**Cum GDD °C**	**Intr rain (mm)**	**Cum rain (mm)**
		**LVS**	**STM**	**HSK**	**EAR**	**KRN**	**COB**					
ACRE 2013	V16							23-Jul	91 (6)	807	34	248
Planted	^**^ER1							23-Jul	60 (4)	807	34	248
May 14th	R1							31-Jul	36 (5)	869	2	252
	R2							5-Aug	51 (5)	921	21	269
	R4							23-Aug	48 (4)	1109	1	294
	R5							14-Sep	63 (5)	1378	24	332
PPAC 2013	V14							2-Aug	32 (4)	624	5	345
Planted	V16							7-Aug	58 (6)	672	45	346
June 5th	R1							12-Aug	56 (5)	728	34	380
	R2							16-Aug	31 (4)	759	11	391
	R4							17-Sep	32 (5)	1103	4	428
	R5							10-Oct	44 (5)	1271	66	556
ACRE 2014	V15							8-Jul	63 (5)	721	4	230
Planted	R1							21-Jul	49 (5)	851	0	274
April 25th	R2							5-Aug	66 (5)	1004	32	325
	R4							22-Aug	66 (4)	1141	13	380
	R5							2-Sep	92 (6)	1269	42	443
	R1R6							25-Sep	727 (66)	1535	318	592

*Growth stages of ^15^N application and subsequent biomass harvesting and biomass partitioning in each stage throughout the growing season. Calendar dates of biomass harvesting (Biomass Harvest date) after 4 to 6 days of ^15^N application. Interval of cumulative growing degree days from ^15^N application to biomass harvesting (GDD°C) and number of days from ^15^N application to biomass harvesting in parentheses (^15^N days). Cumulative growing degree days from planting to biomass harvesting of each growth stage (Cum GDD°C). Interval of cumulative rainfall in millimeters from ^15^N application to biomass harvesting in each growth stage (Intr Rain mm). Cumulative rainfall in millimeters from planting date to biomass harvesting in each growth stage (Cum Rain mm)*.

* *LVS Leaf; STM Stem; HSK Husk; KRN Kernel; COB Cob*.

** *EARLY R1 (ER1) represents 50% of plants were at silking when ^15^N was applied, and R1 represents 90% of plants were at silking when ^15^N was applied*.

*Black shaded areas represent the partitioning of plant components at each growth stage*.

The two N rate treatments were 0 (0N), representing soil indigenous N pool and high N stress, and 112 kg N ha^−1^ (112N) as a moderate level of stress; the latter was sidedressed at the V4 stage between corn rows with a DMI Nutri-Placr at 10–12 cm soil depth as Urea Ammonium Nitrate (UAN) (28-0-0). The 0N plots received the same machine pass to avoid variations in plant growth performance due to soil compaction.

Comparisons were made between four modern hybrids varying in NUE with a similar 114 relative maturity range (Dow AgroSciences, Inc., Indianapolis, IN) and one historic hybrid from the 1970's. In this paper, results will be presented as combined means of genotypes to place emphasis on the “multi-stage pulse labeling” technique; a future paper will focus on the methodology's ability to separate hybrid differences.

### Statistical analysis

At all sites, the plot arrangement was a split-plot experimental design, consisting of six replications and two treatment factors. Nitrogen rates were the main plots and hybrids were the sub-plots. The experiment consisted of six replications (because of cost constraints and small plot size, only three replications were used at a time for ^15^N application).

Statistical analyses were executed with SAS GLM (SAS Institute, 2014) at each growth stage and each year separately. The whole unit error (N rate) was pooled with the subunit error (Hybrid) for all ANOVAs because it was not significant for the majority of analyses (*p* > 0.25). A combined location analysis of variance was performed in 2013 for each variable for all growth stages where the error variances were homogeneous for the majority of the stages (*p* > 0.01). Data presentation in this publication is confined to the combined means of both locations in 2013 (as there were no significant treatment differences between locations in a majority of the observations), and the mean of ACRE in 2014. Location by treatment interactions were pooled with the experimental error when not significant (*p* > 0.01). Fisher's protected least significant difference (LSD) test was used to compare treatment means for each growth stage where the corresponding ANOVA *F*-test was significant (*p* ≤ 0.05).

Differences between growth stages were analyzed with Tukey's HSD test (*p* ≤ 0.05) using the R program (R Development Core Team, 2005).

### Method of ^15^N application and biomass harvesting

Near the beginning of the growing season, single-row ^15^N microplots were identified in three replications and were comprised of five (2013) or six (2014) consecutive plants at the appropriate density with sufficient undisturbed border plants (i.e., beyond 1 m from the microplot) so as to maintain a uniform canopy surrounding each plant group.

Microplots were labeled with 3.17 kg of ^15^N ha^−1^ in 2013 and 2.12 kg of ^15^N ha^−1^ in 2014 as Ca (^15^NO_3_)_2_ containing 98 atom % ^15^N (SIGMA-ALDRICH Co., St. Louis, MO). The labeled fertilizer provided 0.041 or 0.027 g of ^15^ N plant^−1^, respectively, for 2013 and 2014 growing seasons which represents a very small portion relative to the main N rate of 112 kg ha^−1^ (equivalent to 1.42 g of N plant^−1^). The ^15^N rate was lowered in 2014 to reduce costs after we learned that we could detect ^15^N concentration differences in all plant components during the entire 2013 season. Our ^15^N rates were similar to the 3.0 kg of ^15^N ha^−1^ applied by Ta and Weiland (1992) with a much lower plant density of ~60,000 plants ha^−1^.

Injection of ^15^N occurred in multiple plant development stages (from late vegetative stage to near the end of the grain filling period (Table 1). Prior to each time of ^15^N application, the labeled fertilizer was diluted in water (~300 ml of water per gram of Ca (^15^NO_3_), placed in 30 ml plastic syringes, properly sealed, and taken to the field. In the field, screwdrivers were used to make holes ~15 cm deep into the soil at a distance of 15 cm perpendicular to the row on both sides of each plant in the microplot. The ^15^N was injected into the hole.

Immediately after the ^15^N application, PVC pipes (0.6 diameter and 30 cm length) were installed on top of each hole and 0.8 l of water was applied (for a total of 1.6 l per plant). The added water was intended to ensure immediate availability of labeled nitrate-N to the plant roots.

Application dates for ^15^N during the late vegetative stages were determined based on when 50% of plants in the entire experiment had fully expanded leaf collar for each targeted stage. Application timing during reproductive stages occurred when over 50% of plants were in early R1 (ER1) (50% silking), R1 (90% silking), R2 (kernel blister), R4 (kernel dough), R5 (kernel dent) (Abendroth et al., 2011). Development stages of biomass harvesting varied slightly among locations and years (Table 1). In general, development stages refer to the time of plant biomass harvest 4–6 days after the ^15^N application (except for the V16 stage at ACRE in 2013 that indicates the time of ^15^N application).

At ACRE in 2013, plants treated with ^15^N at the V16 stage were in the field for 6 days (from ^15^N application 17 July to biomass harvesting 23 July) and reproductive development was so rapid that 90% of plants in the entire experiment had completed silking by the time of biomass harvest. Plants treated with ^15^N at the ER1 stage were harvested 4 days later (23 July). Subsequently, the microplots labeled as R1 received ^15^N when 90% of plants in the experiment had completed silking. For PPAC in 2013, we achieved better separation of the growth stages close to flowering (in part because of cooler temperatures). Thus, because the V16-stage samples at ACRE also covered the tassel formation period we decided it was still appropriate to combine the V16 results at ACRE with the V16 at PPAC. However, ER1 stage was evaluated only at ACRE and the R1 stage represents the combined means for ACRE and PPAC in 2013.

For both locations in 2013, the six actual biomass harvesting times and further ^15^N evaluations were generally completed at the same growth stages except that the V14 harvesting only occurred at PPAC and the early R1 harvesting occurred only at ACRE. Therefore, the 2013 results from V16, R1, R2, R4, and R5 stages consistently represent the means of the two locations (Table 2). In 2014, when there was only one location (ACRE), one of the pre-R1 stage treatments was substituted for a duplicate R1 application time that was then not harvested until the R6 stage (Table 1).

**Table 2:** Effects of overall sidedress N rate (0 and 112 kg N ha^−1^) on ^15^N fertilizer recovery (^15^Nrec, kg kg^−115^N applied) and proportional allocation of ^15^N uptake (^15^Np, %) in plant components at multiple development stages in 2013.

**GS**	**N rate (kg ha^−1^)**	**^15^Nrec (kg kg^−1^)**	**LVS ^15^Np (%)**	**STM ^15^Np (%)**	**HSK ^15^Np (%)**	**EAR ^15^Np (%)**	**COB ^15^Np (%)**	**KRN ^5^Np (%)**
V14	0	0.25 b	56	44	----	----	----	----
	112	0.30 a	51	49	----	----	----	----
V16	0	0.41	50	32 b	15 a	----	----	----
	112	0.46	48	37 a	14 b	----	----	----
ER1	0	0.37	47 a	32 b	16 a	----	----	----
	112	0.42	46 b	40 a	13 b	----	----	----
R1	0	0.32 b	39 b	22 b	11	28 a	----	----
	112	0.34 a	42 a	26 a	10	22 b	----	----
R2	0	0.27 b	35 b	19 b	11 a	35 a	----	----
	112	0.34 a	40 a	25 a	9 b	27 b	----	----
R4	0	0.25 b	26 b	13	3	58 a	----	----
	112	0.33 a	33 a	13	3	51 b	----	----
R5	0	0.18 b	17	11	2	70 b	4 a	66 b
	112	0.28 a	16	10	2	72 a	3 b	69 a

*GS, Growth stage; LVS, Leaf; STM, Stem; HSK, Husk; STV, Stover (leaf + stem + husk + cob); Ear = cob + kernel (KRN)*.

*Within a growth stage, means without letters are not significant different from each other (p > 0.05), and means with different letters are significantly different at p ≤ 0.05 level T-test (LSD)*.

Destructive aboveground biomass of maize was collected from the three (2013) or four (2014) middle plants in the previously selected micro-plots from 4 to 6 days after the ^15^N application. One additional micro-plot application was implemented in 2014 whereby plants receiving ^15^N at R1 stage were not harvested until the R6 stage (R1/R6). Plant biomass was removed by cutting at the base of the stems with the use of a lopper. Plants were partitioned into stems + leaf sheaths + tassels (stems), leaf blades (leaves), husks + shank + silks (husks), and either whole ear (prior to R4) or cobs and kernels at R5 in 2013, and at R4, R5, and R6 stages in 2014. Stems were chopped into sections immediately after harvest and leaf removal. All samples were dried for 7 days at 60°C, weighed and coarse ground. Stems, leaves and husks were ground to pass a 2 mm screen and cobs and kernels were ground to a pass 4 mm screen size. Sub-samples were taken to the isotope laboratory to proceed with the mass spectrometry analysis.

### Calculations of ^15^N abundance in plant tissue

In order to estimate ^15^N uptake (^15^Nu) in plant components, N concentration (Nc) and delta ^15^N (δ^15^N ‰) were determined in the mass spectrometer for each sample. Nitrogen concentration represented the amount of ^14^N + ^15^N in the sample. Delta ^15^N (δ^15^N ‰) was the proportional amount of ^15^N in parts per mil in the sample relative to an international isotope standard of Air (0.366 atom % ^15^N) (Shearer and Kohl, 1986; Fry, 2006; Van Cleemput et al., 2008).

Additionally, δ^15^N ‰ is also described as the ^15^N atom% excess and can be calculated in the mass spectrometer through the isotopic ratio (IR =^15^N/^14^N) in the compounds. Absolute amounts of isotopic N cannot be simply estimated due to its atomic unit (Mariotti, 1983). Therefore, the linear relationship between ^15^N contents and δ^15^N values allows accurate estimation based on the isotope ratio (IR) approach in the mass spectrometer (Fry, 2006).

The isotopic ratio in a standard (IRstrd) represents the amount of ^15^N in the standard relative to the ^15^N abundance in the atmospheric dinitrogen (N_2_) defined as 0.366 atom % ^15^N (Faust, 1981; Shearer and Kohl, 1986; Dawson et al., 2002; Fry, 2006; Van Cleemput et al., 2008). The isotopic ratio in the sample (IRsmp) represents the quantity of ^15^N in the sample relative to the amount of ^15^N in a given standard.

The equations used to calculate ^15^Nu in plant components are as follows:

1. The isotopic ratio in the samples (IRsmp) was calculated through the proportional ^15^N abundance (δ^15^N) in the plant samples relative to the ^15^N abundance in the atmospheric dinitrogen (N_2_) defined as 0.0036764 ‰ (IRstrd) (Fry, 2006; Van Cleemput et al., 2008) (Equation 1):
  (1) IRsmp=(δN151000+1)*0.0036764
2. Converting the IR to the absolute number of ^15^N atoms in 100 atoms of the total Nc in the sample. The absolute amount of atom % ^15^N (at^15^N) was calculated by dividing the proportional amount of ^15^N in the sample (IRsmp) by the total N in the sample (IRsmp + 1). (Equation 2):
  (2) atN15=(IRsmpIRsmp+1)*100
3. Total N uptake (Nu) per plant component was estimated per unit area (kg ha^−1^) by multiplying the aboveground plant biomass (DM) in kg ha^−1^ by the N concentration (Nc) for respective plant components (Equation 3).
  (3) $$\begin{array}{r} \text{Nu}=\text{DM}*\left(\frac{\text{Nc}}{100}\right) \end{array}$$
4. Absolute amounts of ^15^N uptake (^15^Nu) in plant components per unit area (kg ha^−1^) were estimated by multiplying Nu by the ^15^N found in respective plant component (Equation 4):
  (4) N15u=Nu∗(atN15100)
5. Proportional ^15^N uptake (^15^Np) for each plant component was estimated by dividing the ^15^Nu for respective plant component by the total plant ^15^Nu (Equation 5).
  (5) N15p=N15uTotalN15u
6. ^15^N recovery per plant component (^15^Nrec) was estimated per unit area (kg^−1^ kg of ^15^N applied ha^−1^) by dividing the total plant ^15^Nu (kg ha^−1^) by the amount of labeled fertilizer applied at 3.17 or 2.12 kg of ^15^N Ca (^15^NO_3_)_2_ per ha, respectively, for 2013 and 2014 (Equation 6).
  (6) N15rec=TotalN15uTotalN15applied

## Results

### Proportional allocation of the total aboveground ^15^N uptake in plant components, and plant ^15^N recovery efficiency, per growth stage

Results on the fate of the recently applied ^15^N fertilizer in the above-ground plant components are presented using three parameters: total ^15^N uptake per unit area (^15^Nu, kg ha^−1^), the proportional allocation of the total ^15^N uptake (^15^Np, %) in each separate plant component, and plant ^15^N recovery efficiency (^15^Nrec, kg kg^−115^N applied) of the total labeled fertilizer applied (3.2 or 2.1 kg of ^15^N ha^−1^) at each application time for 2013 and 2014, respectively. Hybrid differences in ^15^Nu and ^15^Np of individual plant components, as well as ^15^Nrec, for each stage will not be discussed in detail in this paper but the specific results for each hybrid, and the full main, sub and interaction statistics for each stage are available in the Supplementary Tables S3 and S4. Results were averaged across hybrid treatment for each N rate (0 and 112N). Supplementary Figure S1 demonstrates hybrid impacts on ^15^N allocation to plant components at R1 and R5 stages in 2013.

**Figure 1:**
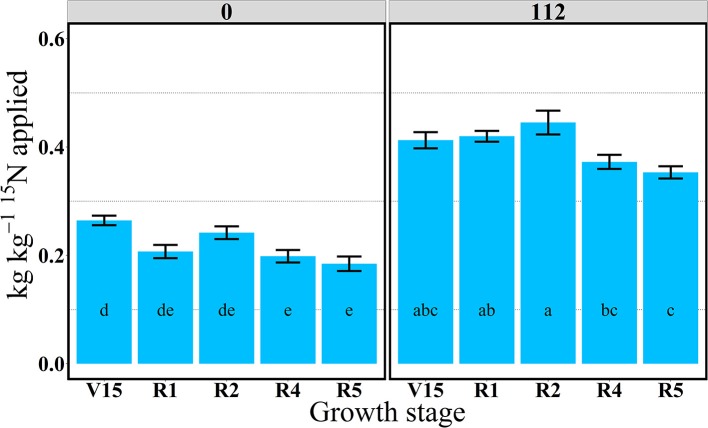
Total aboveground plant ^15^N recovery (^15^Nrec, kg kg^−115^N applied) per growth stage at 0 and 112N in 2013. Values are the means of five hybrids and two locations in Indiana (ACRE and PPAC) in 2013. Total ^15^N applied per growth stage was equal to 3.2 kg ^15^N ha^−1^. Error bars represent the standard error of the means. Letters represent significant differences between growth stages at the 0.05 level (Tukey HSD).

**Figure 2:**
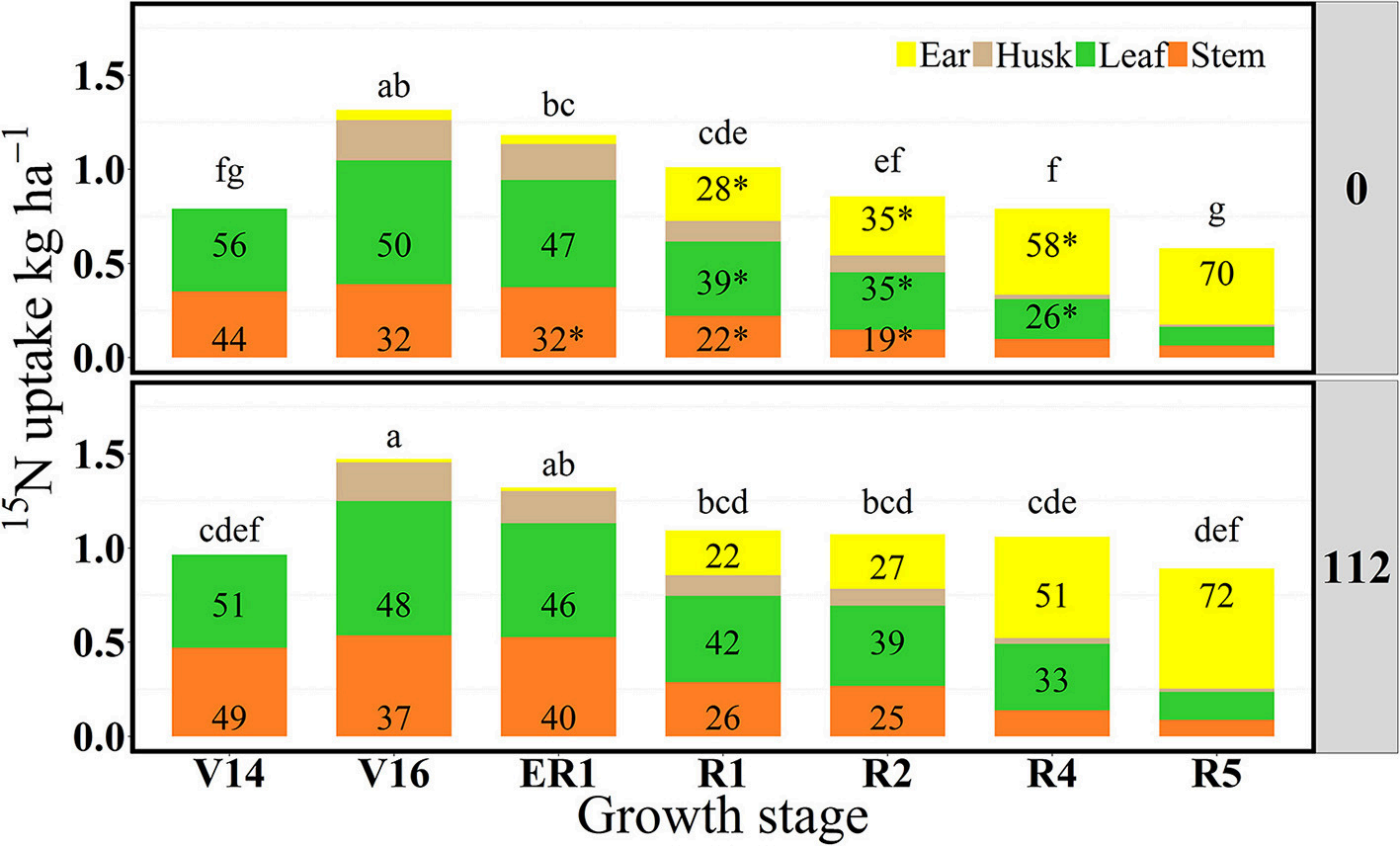
Partitioning of ^15^N uptake (^15^Nu, kg ha^−1^) per plant component (stem, leaf, husk, and ear) and proportional allocation of ^15^N uptake (^15^Np, %) at 0 and 112N over time in 2013. Values are the means of five hybrids and two locations in Indiana (ACRE and PPAC) in 2013. Except for V14 and ER1 representing each location only PPAC or ACRE, respectively. Total ^15^N fertilizer applied per growth stage was equal to 3.2 kg of ^15^N ha^−1^. ^*^Represents significant difference between plant components across N rates at the 0.05 level *T*-test (LSD). Letters represent significant differences across growth stages at the 0.05 level (Tukey HSD).

In 2013, at the V14 stage, mean ^15^Nrec (averaged across five hybrids) represented 0.25 and 0.30 kg kg^−1^ of the total ^15^N applied, respectively for 0 and 112N (Table 2 and Figure 1) and ~50% of that ^15^Nu was accumulated in the leaves and 50% in the stems (Figure 2). The maximum ^15^Nrec within the 6-day period following ^15^N application was observed near the critical period bracketing silking; at the V16 stage plants recovered almost half of the ^15^N applied (~0.45 kg kg^−1^) for both N rates, and ~50% of that ^15^Nu accumulated in the leaves followed by 35% in the stems, 15% in the husks (Table 2 and Figure 2). Only a negligible amount was found in the ear shoots that were just beginning to enlarge (data not shown). Nitrogen rate had no impact on ^15^Np in leaves; however, the 112N treatment increased ^15^Np to stems, while decreasing ^15^Np to husks. At the ER1 stage (when ~50% of plants in the entire experiment had visible extruded silks), maize plants recovered almost 0.40 kg kg^−1^ of the ^15^N fertilizer applied, and of this ^15^N uptake about 48% was allocated to the leaves, 36% to the stems, and 15% to the husks (Table 2 and Figure 2). At this ER1 stage, plants that were stressed due to the lack of N applied at 0N partitioned significantly more ^15^Np to the leaf and husk components than those at 112N (Table 2 and Figure 2). Similar patterns of ^15^Np between V16 and ER1 stages was probably due to overlapping days of ^15^N application and biomass harvesting at the ACRE site. Reducing the time from ^15^N application to biomass harvesting lowered total ^15^Nu at ER1 as expected (Figure 2). By dividing total ^15^Nu by number of days that plants remained in the field (4–6) after ^15^N application, it was observed that there was a similar daily ^15^Nu of ~0.30 kg of ^15^N ha^−1^ day^−1^ between those two stages at ACRE location (data not shown).

In 2013, at the end of R1 stage (i.e., when >90% of plants completed silking), the ^15^Nrec declined, relative to previous stages, to about 0.32 and 0.34 kg kg^−1^ of the ^15^N applied, respectively, for 0 and 112N (Table 2 and Figure 1). At the R2 stage, plants recovered similar amounts of ^15^N as the R1 stage, but more ^15^Nu was partitioned to the ears (35 and 27% at 0 and 112N respectively) at the R2 stage than at R1. Additionally, at the R2 stage leaves and ears received equal ^15^Np in the 0N treatment (~35%). In the R4 stage it was observed that, out of 0.25–0.33 kg kg^−1^ of the ^15^N recovered by plants (at 0 and 112N, respectively), about 58 and 51% of the accumulated ^15^Nu was allocated into the ears, followed by 26–33% to leaves (Table 2 and Figure 2). Although the R5 stage presented the lowest total ^15^Nu of the growing season, plant ^15^Nrec was about 0.18 or 0.28 kg kg^−1^ of the ^15^N applied, respectively, for 0 and 112N rates. Approximately 68% of this late-stage ^15^N uptake was allocated to the kernels, followed by ~17% to the leaves, 11% to stems, and 3% to the husks.

**Figure 3:**
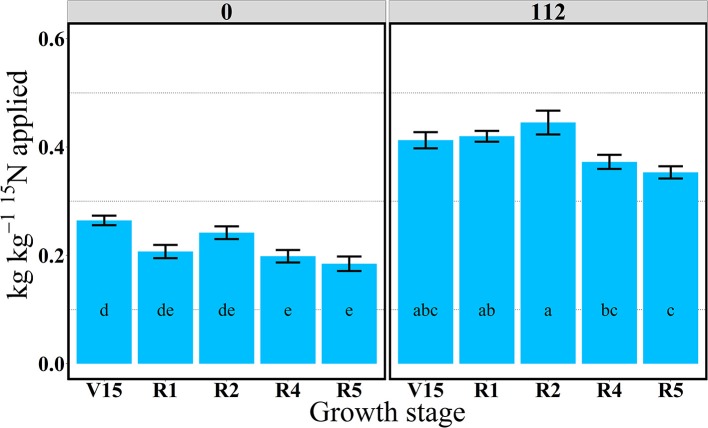
Total aboveground plant ^15^N recovery (^15^Nrec, kg kg^−115^N applied) per growth stage at 0 and 112N in 2014. Values are the means of five hybrids and one location in Indiana (ACRE). Total ^15^N applied per growth stage was equal to 2.12 kg ^15^N ha^−1^. Error bars represent the standard error of the means. Letters represent significant differences between growth stages at the 0.05 level (Tukey HSD).

**Figure 4:**
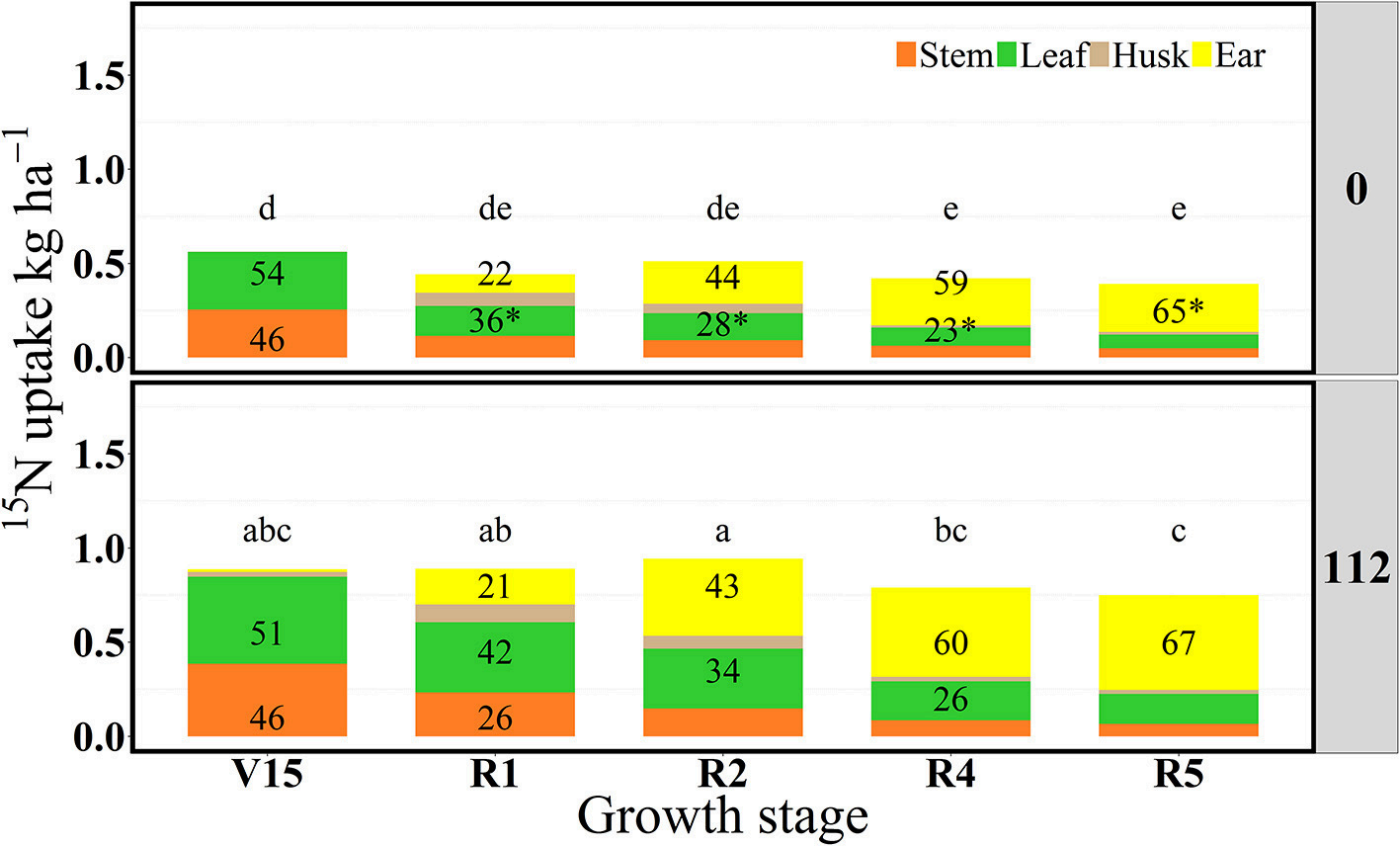
Partitioning of ^15^N uptake (^15^Nu, kg ha^−1^) per plant component (stem, leaf, husk, and ear) and proportional allocation of ^15^N uptake (^15^Np, %) at 0 and 112N over time in 2014. Values are the means of five hybrids and one location in Indiana (ACRE). Total ^15^N fertilizer applied per growth stage was equal to 2.12 kg of ^15^N ha^−1^. ^*^Represents significant difference between plant components across N rate at the 0.05 level *T*-test (LSD). Letters represent significant differences across growth stages at the 0.05 level (Tukey HSD).

In 2014, ^15^Nrec was nearly constant across all growth stages from V15 to R5 stage (Figure 3). There were larger differences across N rates this year than in 2013. At the R1 stage mean ^15^Nrec was about 0.21 and 0.42 kg kg^−1^, at 0 and 112N respectively (Table 3 and Figure 3), and, when averaged over both N rates, ~40% of this ^15^Nrec accumulated in the leaves, 25% in the stems, 11% in the husks, and a significant amount of 23% was allocated to the ears (Figure 4). At the R2 stage, plants recovered about 0.24 and 0.44 kg kg^−1^ of the ^15^N applied, at 0 and 112N respectively, allocating 31% to the leaves, 17% to the stems, and 44% to the ears on average of both N rates (Table 3 and Figure 4). Even with a smaller ^15^N fertilizer rate applied in 2014, plants recovered ~5% more of ^15^Nu at R2 in 2014 than in 2013, and a smaller proportion of ^15^Nu was partitioned to the vegetative components while higher amounts of ^15^Np were found in ears in 2014 than in 2013. In contrast to 2013, at the R4 stage in 2014 ^15^Nu allocation to the ears (60%) was not statistically different for both N treatments. At R5, lower ^15^Np was found in the ears (62%) at both N rates in 2014 than in 2013.

**Table 3:** Effects of overall sidedress N rate (0 and 112 kg N ha^−1^) on ^15^N fertilizer recovery (^15^Nrec, kg kg^−115^N applied) and proportional allocation of ^15^N uptake (^15^Np, %) in plant components at multiple development stages in 2014.

**GS**	**N rate (kg ha^−1^)**	**^15^Nrec (kg kg^−1^)**	**LVS ^15^Np (%)**	**STM ^15^Np (%)**	**HSK ^15^Np (%)**	**EAR ^15^Np (%)**	**COB ^15^Np (%)**	**KRN ^15^Np (%)**
V15	0	0.26 b	54	46	----	----	----	----
	112	0.41 a	53	44	----	----	----	----
R1	0	0.21 b	36 b	26	16 a	22	----	----
	112	0.42 a	42 a	26	11 b	21	----	----
R2	0	0.24 b	28 b	18 a	10 a	44	----	----
	112	0.44 a	34 a	16 b	7 b	43	----	----
R4	0	0.20 b	23 b	15 a	3	59	5 a	54
	112	0.37 a	26 a	11 b	3	61	4 b	57
R5	0	0.18 b	18 b	13 a	3	65 b	4 a	61
	112	0.35 a	21 a	9 b	3	67 a	3 b	64
R1R6	0	0.45 b	6 b	7	4 a	82	7a	75 b
	112	0.72 a	8 a	6	3 b	83	5 b	78 a

*GS, Growth stage; LVS, Leaf; STM, Stem; HSK, Husk; STV, Stover (leaf + stem + husk + cob); Ear = cob + kernel (KRN)*.

*Within a growth stage, means without letters are not significant different from each other (p > 0.05), and means with different letters are significantly different at p ≤ 0.05 level T-test (LSD)*.

For the N rate factor, plant ^15^Nrec was almost always N rate dependent (Tables 2, 3). Furthermore, results suggest a strong influence on both ^15^N accumulation and the ^15^N allocations to the sink and source organs. A higher N rate generally helped increase ^15^Nu by maize plants at all development stages. Even though leaves accumulated higher amounts of ^15^N at the moderate N rate of 112N, before the flowering period in 2013 it was observed that ^15^Np to leaf components was higher with 0N treatment than at 112N. Conversely, both ^15^Np and ^15^Nu in stem components were higher at 112N than with the 0N. In the same year, plants with more N deficiency stress allocated more ^15^N to their reproductive ear organs at 0N than with the 112N rate. Except for the R5 stage, ^15^Np to the ears (cobs + kernels) following ^15^N application during reproductive stages was higher at 112N. In 2014, ^15^Nu accumulation as well as the proportional allocation of ^15^Nu to leaves was higher at 112N than at 0N for the entire season. In contrast to the previous year, the allocation of the ^15^Nu to the stems, husks and cobs was significantly larger at the 0N than with the 112N during the reproductive period.

### Total ^15^N uptake rate per GDD and total aboveground dry matter accumulation per GDD

**Figure 5:**
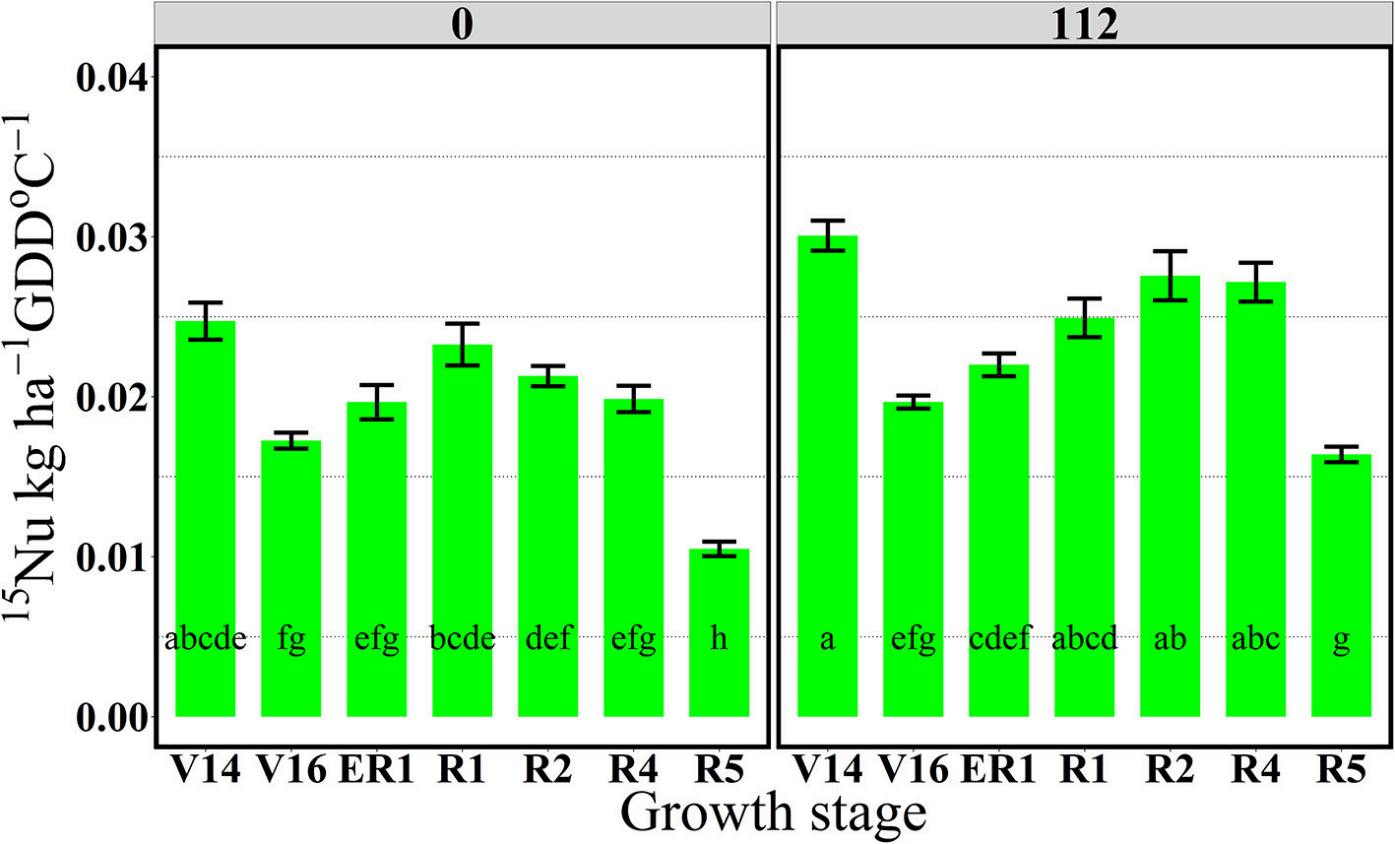
Effects of growth stages on total ^15^N uptake rate per GDD (^15^Nu, kg ha^−1^ GDD °C^−1^) at two N rates 0 and 112 kg N ha^−1^. Values are the means of two locations (ACRE13 and PPAC) in 2013. Total ^15^N applied per growth stage was equal to 3.2 kg ^15^N ha^−1^. Error bars represent the standard error of the means. Letters represent significant differences between growth stages at the 0.05 level (Tukey HSD).

**Figure 6:**
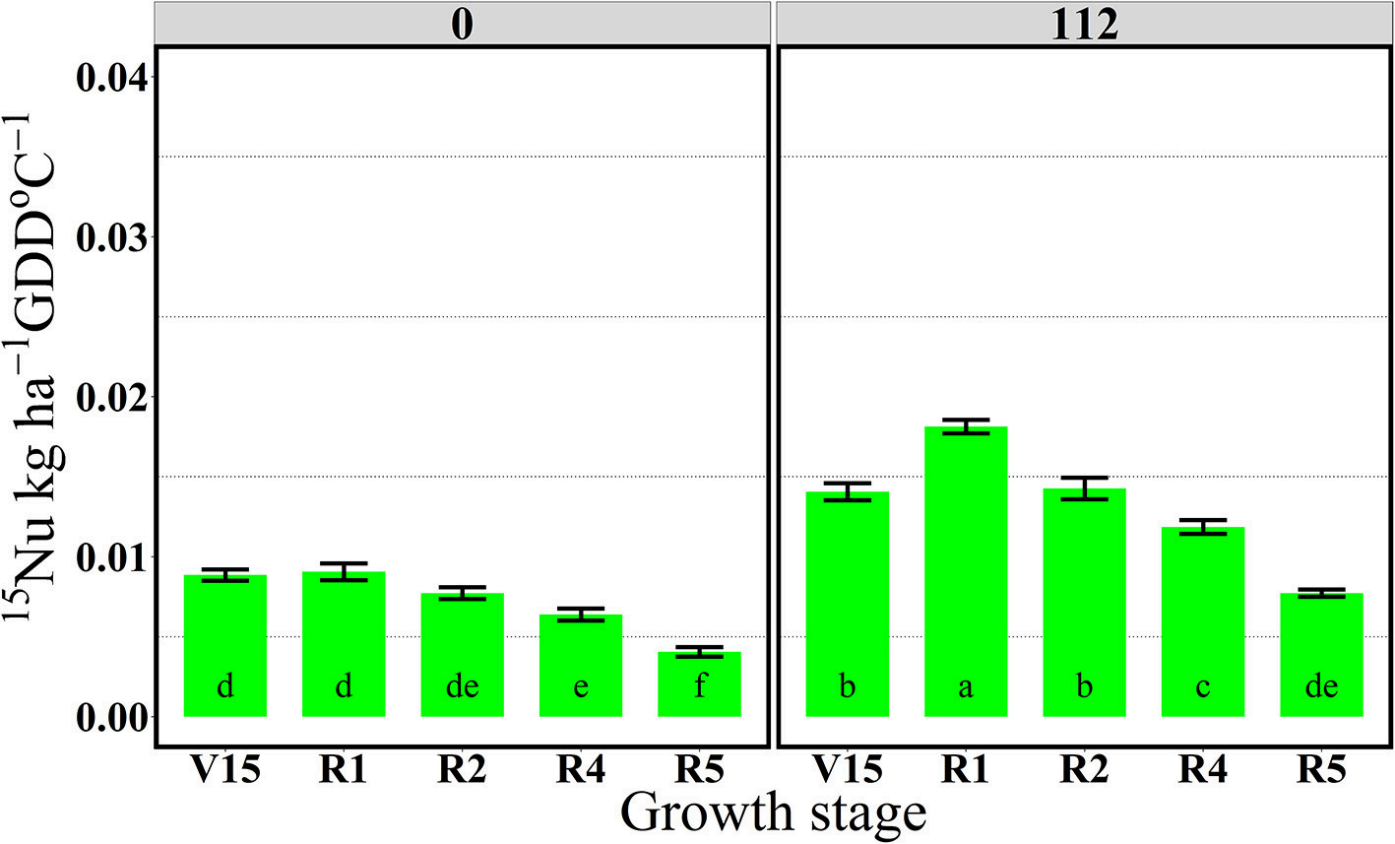
Effects of growth stages on total ^15^N uptake rate per GDD (^15^Nu, kg ha^−1^ GDD °C^−1^) at two N rates 0 and 112 kg N ha^−1^. Values are the means of one location (ACRE) in 2014. Total ^15^N applied per growth stage was equal to 2.12 kg ^15^N ha^−1^. Error bars represent the standard error of the means. Letters represent significant differences between growth stages at the 0.05 level (Tukey HSD).

Through the “multi-stage pulse labeling” ^15^N method it was also possible to estimate the extent to which plants uptake N during the growing season (Figures 5, 6). The amount of ^15^N taken up per daily cumulative GDD was calculated by dividing total plant ^15^Nu (kg ha^−1^) (Tables S1, S2) by the GDD °C (base 10°C) units accumulated from ^15^N application to plant biomass harvest (Table 1).

In 2013, the 112N treatment increased amounts of ^15^Nu per GDD after the flowering period, from R2 to R5 stages, relative to the 0N treatment. However, in 2014, ^15^Nu per GDD was higher at the 112N rate in comparison to the 0N rate for all stages.

For both years, the period of greatest ^15^Nu per GDD occurred from V14 to R4 stage, and the lowest ^15^Nu per GDD was observed at the R5 stage for both N rates (Figures 5, 6). In 2013, although plants showed higher ^15^Nrec at the V16 relative to the reproductive stages (R2, R4, and R5) (Figure 1), the actual rate of ^15^Nu accumulation per GDD was similar across stages, except that ^15^Nu per GDD was much lower at the R5 stage (Figure 5).

**Figure 7:**
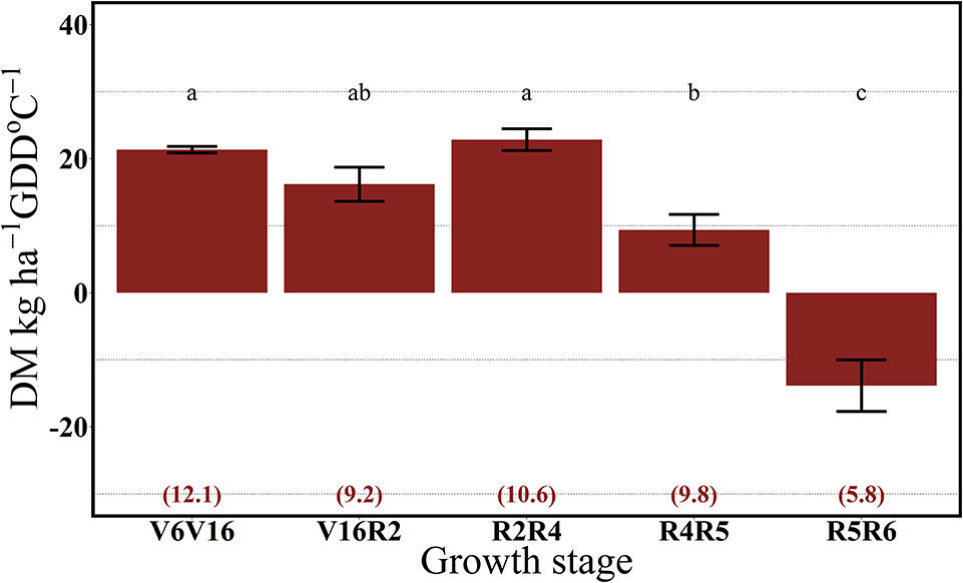
Effects of growth stage intervals on total DM accumulation per GDD (DM, kg ha^−1^ GDD °C^−1^) average of two N rates (0 and 112 kg N ha^−1^). Numbers in parentheses are the daily cumulative GDD per growth interval (GDD °C day^−1^). Values are the combined means of two locations (ACRE and PPAC) in 2013. Error bars represent the standard error of the means. Letters represent significant differences between growth stage intervals at the 0.05 level (Tukey HSD).

**Figure 8:**
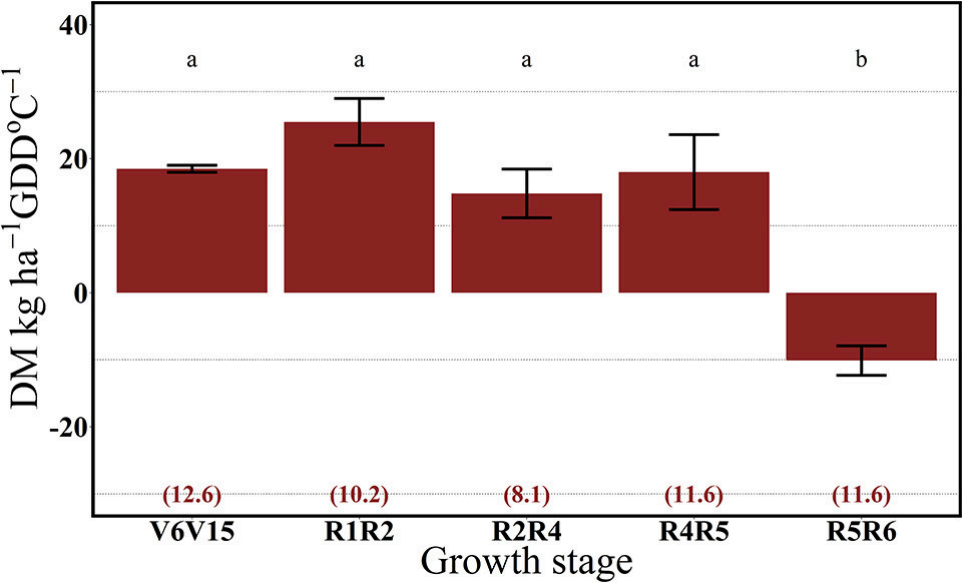
Effects of growth stage intervals on total DM accumulation per GDD (DM, kg ha^−1^ GDD °C^−1^) average of two N rates (0 and 112 kg N ha^−1^). Numbers in parentheses are the daily cumulative GDD per growth interval (GDD °C day^−1^). Values are the means of one location (ACRE) in 2014. Error bars represent the standard error of the means. Letters represent significant differences between growth stage intervals at the 0.05 level (Tukey HSD).

For both years, the 112N rate significantly increased dry matter accumulation (DM) per GDD in comparison to the 0N treatment (Figures 7, 8). There was no interaction between growth stages and N rate (data not shown). In 2013, the highest rate of DM accumulation was observed from V6 to the R4 stage with about 20 kg ha^−1^ per GDD on average of both N rates (0 and 112N) (Figure 7). In 2014, rates of DM accumulation per GDD were relatively consistent from V6 to R5 at ~20 kg ha^−1^ per GDD °C (Figure 8), or for a longer period relative to 2013. Additionally, the apparent lower DM accumulation from R2 to R4 stage could be explained by the lower GDD accumulation during that period.

Our results suggest that plant N uptake is strongly driven by both the sink strength and source availability. In 2013, plants took up similar rates of the ^15^N applied up to the R4 stage and DM accumulation began to decrease from R4 to R5 stages. In 2014, with possibly more acute soil N deficiency, ^15^Nu per GDD decreased even with a continual DM accumulation up to the R5 stage.

### Fate of labeled N taken up at silking and its final allocation at physiological maturity

**Figure 9:**
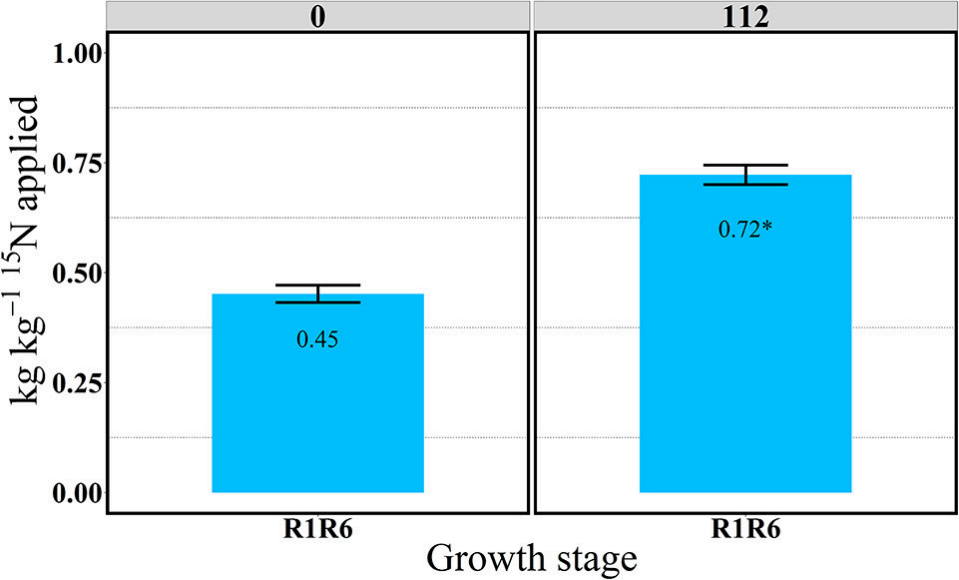
Total aboveground plant ^15^N recovery (^15^Nrec) at the R1R6 growth stage at 0 and 112N in 2014. Values are the means of five hybrids and one location in Indiana (ACRE). Total ^15^N applied per growth stage was equal to 2.12 kg ^15^N ha^−1^. Error bars represent the standard error of the means. ^*^Represents a significant difference across N rates at the 0.05 level *T*-test (LSD).

**Figure 10:**
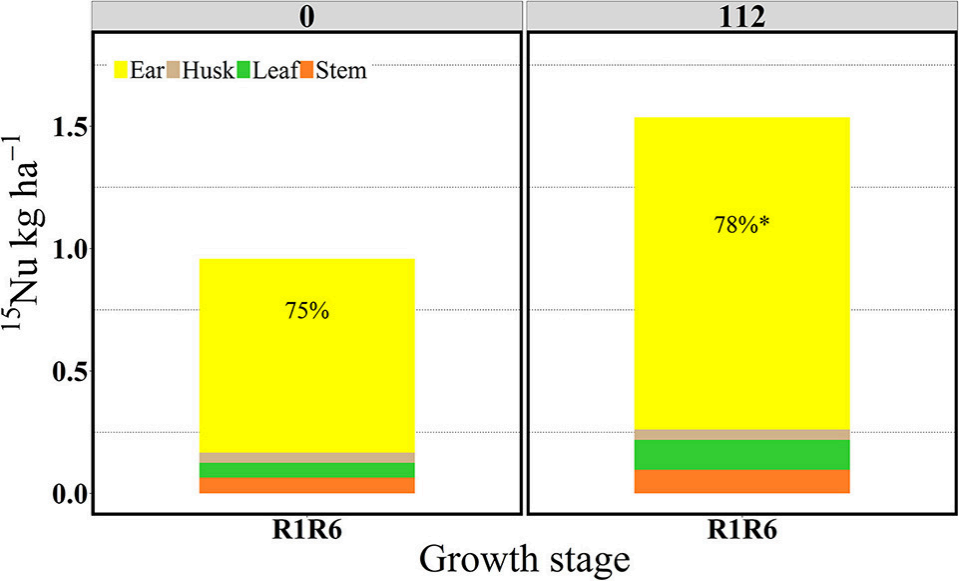
Partitioning of ^15^N uptake (^15^Nu, kg ha^−1^) per plant component (stem, leaf, husk, and ear) and proportional allocation of ^15^N uptake (^15^Np, %) at 0 and 112N at the R1R6 stage in 2014. Values are the means of five hybrids and one location in Indiana (ACRE). Total ^15^N fertilizer applied per growth stage was equal to 2.12 kg of ^15^N ha^−1^. ^*^Represents a significant difference of ear ^15^Np across N rate at the 0.05 level *T*-test (LSD).

In the “long term” labeling method implemented in 2014, the amount of ^15^Nu accumulated during the grain filling period of the ^15^N applied at silking (R1) was measured at physiological maturity in the duplicate R1R6 microplots (Table 3 and Figures 9, 10). At maturity ^15^Nrec was dramatically affected by the sidedressed N treatments; plants recovered ~0.45 and 0.72 kg kg^−1^ of the ^15^N fertilizer applied at R1, respectively, for 0 and 112N (Figure 9). Although the proportional allocation of the ^15^Nu (^15^Np) in the whole ear (cob + kernel) was not affected by the N rate, more ^15^Np to the kernels was observed with 112N (Table 3 and Figure 10). Overall, averaged over both N rates, ~77% of that accumulated ^15^N uptake at flowering (from the ^15^N application at R1) was partitioned to the kernels at maturity, followed by ~7% to the leaves, stems, and cobs and 4% to the husks. In the pulse labeled method, total plant ^15^Nrec averaged ~0.21 and 0.42 kg kg^−1^ of the ^15^N applied in the ~5 day period at R1; thus the remaining ^15^Nrec (0.24 and 0.30 kg kg^−1^) that was found at R1R6 was the ^15^Nu that was recovered during the rest of the grain filling period.

We also used the net change approach to calculate the post-flowering ^15^N uptake per unit area (post^15^N, kg ha^−1^) as (Total plant ^15^Nu at R6 minus Total plant ^15^Nu at R1), to estimate remobilized ^15^Nu per unit area (^15^N rem, kg ha^−1^) as (Total plant ^15^Nu at R1 minus Stover ^15^Nu at R6), and to estimate the ^15^N remobilization efficiency (^15^NremEF, kg kg^−1^) as (Total plant ^15^Nu at R1 minus Stover ^15^Nu at R6 divided by Total plant ^15^Nu at R1) (Table 4). Stover was the sum of all plant components except kernels. Because we estimated the ^15^Nu at the silking period (multi-stage pulse labeling method) (Table 3), it was also possible to differentiate the proportion of the ^15^Nu in the grain that originated from the ^15^Nu accumulated during the reproductive period [i.e., post-flowering ^15^N uptake (post^15^N)], to the amount of ^15^Nu remobilized (^15^Nrem) from silking to maturity (Table 4).

**Table 4:** Consequences of overall N rate on ^15^N dynamics at grain maturity (R6 stage) following ^15^N application at the silking period (R1 stage) in 2014.

**N rate (kg ha^−1^)**	**^15^Nrem (kg ha^−1^)**	**^15^NremEF (kg kg ^−1^)**	**^15^NremGr (kg kg ^−1^)**	**^15^Nrem/Ttl^15^Nu (%)**	**Post^15^N (kg ha^−1^)**	**Post^15^NuGr (kg kg ^−1)^**	**Post^15^N/Ttl^15^Nu (%)**
0	0.21 b	0.45 b	0.29 b	22 b	0.52 b	0.71 a	54 a
112	0.56 a	0.63 a	0.47 a	36 a	0.64 a	0.53 b	42 b

*Means with different letters are significantly different at p ≤ 0.05 level T-test (LSD)*.

Pan et al. (1986) calculated Post^15^N uptake (Post^15^N) in maize using the net change approach; however, they sampled plants at the R1 stage on the same day that ^15^N was applied for the “long term” evaluation. Therefore, it might not be possible to estimate post^15^N and ^15^Nrem by the net change approach in our study since we applied ^15^N at the same time for both pulse labeled and long term approaches. Thus, from this method of calculation, the post^15^N represented about 54–42% of the total ^15^Nu accumulated from R1 to R6, and the ^15^Nrem accounted for 22–36% of the total ^15^Nu at R6, respectively, for 0 and 112N treatments. For both sources of ^15^Nu to the grain it was found that post^15^N contributed more at 0N than with 112N. Post ^15^N averaged ~70 and 53% of the grain ^15^Nu at 0 and 112N, respectively, while ^15^N rem contributed ~30–47% to grain ^15^Nu at R6, respectively, for 0 and 112N. The higher post^15^NuGr at 0N that was calculated by the net change from R1 to R6 may be explained by the larger effect of the N rate on the actual quantities of ^15^Nu.

## Discussion

### Use of “multi-stage pulse labeling” ^15^N technique to determine the fate of the recent ^15^N uptake into plant components

In contrast to most studies using labeled N, this research describes the potential implementation of the “multi-stage pulse labeling” ^15^N application in field-grown maize; this approach involves tracking ^15^N uptake and identifying changes in the proportional allocation of the recent ^15^N taken up into plant components at multiple maize development stages when field maize is grown under limited N supply. It is well-known that plants with superior ability to remobilize N to sink components yield more (Cliquet et al., 1990a), and that the higher ability of modern hybrids to continue accumulating N after the flowering period with efficient N allocation to its sink organs has increased both NUE and yield (Ciampitti et al., 2013). For these reasons, we regard the use of the labeled N technique to accurately estimate the fate of the recent N uptake in specific plant components over time, and especially during reproductive growth stages, to be a potentially powerful phenotyping tool.

For this 2-year study using the “multi-stage pulse labeling” technique, and after combining means of five hybrids, at two N rates (0 and 112N), it was observed that prior to flowering the distribution of short-term ^15^N uptake to leaves and stems was not affected by the N rate treatment as much as the actual amount of ^15^N uptake (except for V16 stage in 2013). Paponov and Engels (2005) also observed no effect of N rates (873 and 2,626 mg of N per plant) on ^15^N allocation to plant components during the vegetative and reproductive period. In our study, at late vegetative stages, maize plants primarily allocated ^15^Nu to their main sinks such as leaves and stems followed later by the husks and immature earshoots. Approximately 50% of the total ^15^N uptake was allocated to leaves for both N rates (0 and 112N). In contrast to our results, Cliquet et al. (1990b) analyzed N allocation in the below and aboveground maize plant components (8 days after ^15^N application) and found that stems were a stronger sink component than all plant organs at late vegetative stage; out of the total plant new N uptake, stems accumulated ~45% of the recent N taken up, followed by the leaf blades (22%) and roots (18%). For both N rates in our study, stems and husks appeared to act as transitional destinations of the ^15^Nu, and ears became an increasingly powerful sink component. Stems appeared to largely function as a “pipeline” where the ^15^N passed through accumulating primarily into the leaves from V14 to ~R2 stage at first and then to the ears at ~R2 or R4 and R5 stages. Ma et al. (1998) also confirmed the movement of the ^15^N to major sink component around the silking period. In their study, 3 days after ^15^N application (by stem infusion) at silking, plants accumulated ~40% of the new N in reproductive organs vs. 30% to the leaves and ~18% to the stems. However, in contrast to our plant partitioning procedure, they included husks as a reproductive organ (which may have resulted in their higher allocation values to reproductive plant parts at R1).

During the grain filling period, the moderate N rate (112N) in our study helped to maintain N contents in maize plants longer in the season, especially in the leaf components, while the 0N treatment showed the dynamics of ^15^Np in plant components deficient in N. Thus, our moderate N supply (112N) increased absolute amounts of ^15^Nu in the plants but it did not necessarily increase the allocation of ^15^Nu to the sink organs. Maize plants increased the allocation of the most recent ^15^Nu to the developing organs under the 0N rate compared to the 112N rate. Likewise, Paponov and Engels (2005) acknowledged the sink power of the ears over the vegetative plant parts at low N rates.

In agreement with our study, Rajcan and Tollenaar (1999) examined the effects of source: sink ratio on the N uptake during the reproductive period and demonstrated that after flowering the accumulation of N gradually declined in the vegetative plant parts with more severe declines occurring at lower N rates, while N accumulation increased into the ears. Our results show that the primary destination of the most recent ^15^Nu at both N rates was similar during the vegetative period but higher to the leaf component at R1 (~42%) and R2 stages (~38%), then increased to the ears at R4 (~55%), and kernels at R5 stages (~70%). The latter overall pattern of N partitioning in the plant was also confirmed by other authors using different labeling approaches. Crawford et al. (1982) provided ^15^N to the plants by nutrient solution just once at the time of pollen shed followed by multiple harvests. Yoneyama et al. (2003) used a leaf feeding labeling method to assess N assimilation in several crops including maize. According to the latter research, in general, N transportation within plants is driven by the two elements of growing plant components and reserve organs, and once N is absorbed in the reserve organs and mature leaves it will probably be reallocated to a developing organ.

The main N treatment influenced the amounts of plant ^15^N uptake and allocation of ^15^N at all growth stages; however, it did not alter the overall vegetative distribution of the ^15^Nu within the plants, and leaves always accumulated more ^15^N than stems. The multi-stage pulse labeling N approach measured the distribution of the recent N uptake by the plants, demonstrating that even at late grain fill plants were actively taking up N and mainly allocating the most recent N to the ears and/or kernels. Therefore, comparison between N treatments in this study was crucial to observe plant response to the N supply until late in the season and also to examine how plants change allocation patterns when they are source limited or stressed due to lack of N. For both N treatments, the largest amount of ^15^Nu and ^15^Nrec within 4–6 days of application was observed at the V16 stage and the lowest at the R5 stage. However, rates of ^15^N uptake per GDD°C were fairly consistent across stages, and only declined at R5. Correspondingly, ^15^Nrec was as high as 0.46 (kg kg^−1^) at 112N to as low as 18 (kg kg^−1^) at 0N. The latter suggests that further investigations in late vegetative stage or late reproductive stages may help to identify plant processes related to its N uptake efficiency.

Our results indicate that plant organs with lower Nc showed higher ^15^Np to the same component, and therefore ^15^Np in the plants operated as a signal of plant organs with the most deficiency in Nc. Paponov and Engels (2005) also found the same tradeoff with higher allocation of the ^15^Np to grain with reduced grain Nc under low N supply. Detailed evaluations of the proportional allocation of the ^15^Nu in plant components in our study suggested the late vegetative stage (V15) is a good stage to indicate relative plant total N status and variation in genotypic responses (data not shown). Indeed, ^15^Np to the leaves at V15 was strongly correlated with the responsiveness of ^15^Np to N rate treatments for the same plant component later in the season (data not shown).

In summary, for both years, leaves were the main sink component up to the kernel set period (R2) and, from R4 stage onward, more than 50% of the ^15^N absorbed accumulated in the ears. However, lower soil N availability and, consequently, higher N stress in 2014 prompted higher partitioning of ^15^Np to ears already at the R2 stage. Plant components that demonstrated higher ^15^Np had more apparent N deficiency.

### Implementation and considerations of ^15^N use in maize field experiments

There are many important factors that should be taken into account for a successful field study using a labeled N approach. Adequate plant sample size in field experiments using labeled N is crucial to identify genotypic responses for ^15^Nu partitioning to plant components. In a greenhouse study, Pan et al. (1986) addressed the large plant biomass variation in total N concentration or N content determination in plant components of five plants harvested at silking. Our studies were based in the field, and microplots were stringently selected for equal-density representations from the middle 3 or 4 plants. In 2014, after increasing sample size to four plants per growth stage and decreasing the ^15^N fertilizer rate by a third, we were still able demonstrate efficient assessment of ^15^Nu dynamics in the plants. Although harvesting more plants is even more preferable, resource costs of labeled N application and analyses are significant barriers.

The experiment layout and distance of plant zones that are used to analyze labeled or unlabeled treatments is critical (Van Cleemput et al., 2008). Several authors have suggested the use of plastic films, chambers, or tarpaulin materials to avoid problems with ^15^N percolation and with cross-contamination among treatments (Anhar, 2005). In the present study, we tried to represent the natural soil conditions in open environments using microplots within the main plots with no physical barrier. Microplots were installed at least 1 m from each other; the distance and the very low rates of ^15^N we utilized helped to circumvent contamination of plant zones by labeled N from elsewhere, and also helped prevent growth rate distortions arising from the extra sunlight in remaining plants harvested for biomass in other zones at a later time. The labeled N was carefully applied into the soil close to the root zones (~15 cm) to avoid direct contact of the ^15^N applied with the plant canopy and that, plus the water application, ensured immediate access of the labeled nitrate to growing roots.

The source of the labeled fertilizer used in the present study [Ca (^15^NO_3_)_2_ 98 atom%^15^N] was intended to make nitrate-N (${\text{NO}}_{3}^{-}$-N) immediately available to the plants and to minimize possible interactions of ammonium N sources (${\text{NH}}_{4}^{+}$-N) to the negatively charged soil colloids. Roots can assimilate N by different mechanisms depending on the N source (Yoneyama et al., 2003), and several studies have observed that maize does not have a preferential uptake between mineral N sources (${\text{NO}}_{3}^{-}$/${\text{NH}}_{4}^{+}$-N) (Reddy and Reddy, 1993; Crozier et al., 1998). However, Pan et al. (1986) found higher accumulation of the labeled (NH_4_)_2_SO_4_ surface applied at planting in the grain when compared to the labeled Ca(^15^NO_3_)_2_ source which was applied 26 and 52 days after planting. Our study assumed that all labeled N taken up by plants was distributed to a uniform depth on both sides of the maize plants with the expectation that N flux into plant components over time should be consistent for the 3 or 4 consecutive plants that were removed from the 5- to 6-plant micro-plot zones.

The amount of labeled fertilizer applied per plant [0.16 g (2014) or 0.24 g (2013)] was enough to enable instrument detection of the ^15^N into all plant components, and yet not so much that it changed the dominant soil plus fertilizer N supply pool that met the majority of the maize plant N requirements. The “spike” of the highly enriched labeled fertilizer injected into the soil allowed a precise ^15^N determination in the mass spectrometer. The additional water after each time of ^15^N application was crucial to increase the N availability to plants via mass flow while reducing the risk of soil-mineral interactions from the fertilizer ^15^N applied with soil microorganisms. Labeled N (^15^NO^−3^) losses via leaching were expected to be minimal with the 1.6 l of water applied unless there was an intensive rain event between the pulse application and biomass harvest.

In the isotope laboratory, highly enriched plant materials required the use of additional “blank” and standard samples to monitor and avoid ^15^N carryover between labeled materials during the mass spectrometer analysis. Thus, lower quantities of labeled fertilizer were applied in 2014 because we realized after the first year that extremely enriched plant samples increased the risk of subsequent sample contamination during testing. High costs (i.e., >$35,000 with the labeled fertilizer alone in the 2-year period) made it even more imperative to utilize a lower practical rate of ^15^N in the second year.

The natural abundance of isotopic N in the atmospheric dinitrogen (N_2_) is considered equal to 0.366 atom %^15^N in a ratio (^14^N:^15^N) equal to 273:1 (Boaretto et al., 2004; Schepers and Raun, 2008). Similar values were found in the present study by sampling control plants in all hybrid and N rate treatments at the R4–R5 growth stages. These “control” values were used to calculate atom %^15^N excess (At%^15^NExcs) in plant components for each labeled sample as the amount of ^15^N which exceeded that represented in the non-labeled plant tissues originating from the natural abundance in the soil. The At%^15^NExcs was estimated by subtracting atom%^15^N (At^15^N) of each labeled plant component by the N natural abundance found in the present study (0.3683 or 0.3677 Atom %^15^N) (Equation a). In this work, amounts of pre-existing ^15^N in the soil based on natural abundance were neglected; however, the latter approach confirmed that soils were not labeled before the current field experiment. Additionally, discrimination from the uptake of natural abundant ^15^N to the ^15^N fertilizer was not important to estimate in our evaluations of short-term ^15^N accumulation in the plants because the large 98% enrichment of the fertilizer ^15^N applied provided confident identification of the isotope N taken up by plants.

(Eq.a) $$\begin{array}{r} \text{At}{\%}^{15}\text{NExcs}={\text{At}}^{15}\text{N}-0.3683 \end{array}$$

### Perspectives on labeled ^15^N approaches in field maize research

The use of isotopic N has been acknowledged for its accuracy as N tracer for several decades (Van Cleemput et al., 2008). The multifaceted use of ^15^N in open systems is, when properly conducted, highly effective for N balance investigations in real, as distinct from simulated, soil-plant systems under different G × E × M interaction treatments. In this work, through mass spectrometry analysis, we estimated the δ^15^N in aboveground plant components to calculate uptake, partitioning, allocation, and recovery of N throughout the season. Observations of high correlations between ^15^N and total N allowed for detailed evaluations of maize plant response to labeled N additions in the root zone at multiple stages of development and at different N rates. After decades evaluating variations of plants δ^15^N in both laboratory and field experiments using ^15^N feeding methods, Yoneyama et al. (2003) also showed a strong association of δ^15^N with N dynamics in several crops.

Several techniques have been implemented using ^15^N in either open or controlled environments (field, greenhouses, and chambers) during short- or long-term methods of ^15^N application (Duete et al., 2009; Ning et al., 2017). However, few studies have invested in field studies using highly enriched ^15^N to address the allocation of N in plant components spanning the reproductive period or other intervals of the growing season in maize.

The “long term” labeled N application has occasionally been implemented as a one-time alternative to differentiate and more precisely estimate post-flowering N uptake and N remobilization from late vegetative stage or silking periods to physiological maturity. This method has helped to quantify N flux within the plants over the period of interest. Mae and Ohira (1981) and Ta and Weiland (1992) calculated the ^15^N remobilization by subtracting the ^15^N uptake in the stover at R6 by the ^15^N in the stover at R1. However, ^15^N remobilization can also be estimated as the difference between the ^15^N accumulated in the stover at maturity minus the whole plant ^15^N uptake at silking (Pan et al., 1986). The latter calculation was used in the present study.

When we compared ^15^Nu accumulated in the R1R6 zones by the more traditional “long term” evaluation method of ^15^N application vs. the results of the Pan et al. (1986) study, we observed a much higher level (~77%) of ^15^Nu accumulated in the kernels at R6 vs. the lower allocation (~51%) of ^15^N uptake to the grain in the Pan et al. study (Table 3). This may be partially explained by their lower total plant ^15^N recovery (~75%) when compared to ours (~83%), by their use of older and different genotypes that preferentially accumulated ^15^N to the stalk and root components, and by their shorter post-flowering period of 33 days in comparison to our 66 days. Furthermore, Pan et al. (1986) found that post ^15^N accounted for 42% of the total ^15^Nu accumulated in the plant at physiological maturity, while we found that post ^15^N contributed 54 and 42% to the total ^15^Nu accumulated at R6, for 0 and 112N treatments (Table 4). Similarly to our results, the review study of Ciampitti and Vyn (2013) demonstrated that post-flowering N uptake can contribute up to about 56% of the total plant N uptake at R6 stage.

Results from Ma et al. (1998) generally agreed with our current findings for the “long term” ^15^N accumulation. While we found that 77% of ^15^Nu in the kernels was taken up from R1 to R6 stage, their results from a one-time ^15^N application show 65–75% of infused ^15^N was accumulated in the kernels. However, they estimated 59 and 82% as the amount of remobilized N going to the grain from vegetative components, and a much lower contribution of the post-silking ^15^N uptake to the total ^15^Nu at R6 (ranging from 18 to 41%, which was similar to our 42% in the 112N treatment). Bertin and Gallais (2000) indicated a range of 35–65% of the N accumulated in the grain originated from N remobilization. We observed that remobilized ^15^N accounted for 22 and 36% of the total ^15^Nu accumulated at R6, for 0 and 112N rates (Table 4).

Furthermore, in attempting to integrate the isotope technique and breeding approaches, Gallais et al. (2007) estimated the contribution of the N remobilization and post N uptake in several maize inbred lines using a one-time ^15^N application. In their 3-year experiment, comparing different methods of labeled N application, they found that ~83% of that ^15^Nu was accumulated into the kernels at R6, slightly higher than our ^15^N proportional allocation ~77%. They also estimated ~25% of the total ^15^Nu at maturity originated from the post-flowering N uptake and about 62% was coming from the ^15^N remobilization. Our study found that post ^15^N accounted for ~50% of the total ^15^Nu accumulated at R6, and about 30% of that ^15^N accumulated at R6 originated from the remobilized ^15^N (Table 4). The difference in results of these two detailed studies may be explained by our ^15^N application at silking while their ^15^N application was at the beginning of stem elongation period (which enabled a better estimate of the accumulation in vegetative components), and by their use of ^15^N fertilizer with much lower enrichment to test an even larger number of genotypes.

This “long term” labeled approach has been broadly used to assess ^15^N uptake dynamics at multiple plant development stages (Friedrich and Schrader, 1979; Cliquet et al., 1990a,b; Deléens et al., 1994; Ma et al., 1998; Ning et al., 2017). When the influence of the ^15^N application at planting and at anthesis on improvements of N harvest index (NHI) in wheat (*Triticum aestivum* L.) was studied, Wuest and Cassman (1992) showed that ^15^N applied at flowering improved NHI (0.89) by almost 20%. The latter suggests a possibility that the ^15^N injection at the R1 stage overestimates the real amounts partitioned to the grain during the reproductive period.

Apart from injection method into the soil, Hertenberger and Wanek (2004) suggested that ^15^N stem infiltration and leaf feeding were the most effective labeling techniques to analyze N in thick-stem plant and grasses species, respectively. More recently, Putz et al. (2011) has advanced the termed “*in-situ*” labeling technique in plant species cultivated in greenhouse. Through the use of paint brush, they applied 2 mg of ^15^N Urea (98 atom% ^15^N) onto the leaves once a day during 5 days and plants were collected at short interval of hours after labeling.

Hence, in contrast to the techniques previously described by authors as “long term” and “short term” labeling approaches, our modified “multi-stage pulse labeling” technique ^15^N was used as a tracer to evaluate the fate of recently applied ^15^N into maize plants. The “multi-stage pulse labeling” technique used very small absolute amounts of highly enriched ^15^N fertilizer (98 atom% ^15^N) and different plants were routinely exposed to the labeled N for short intervals (just 4–6 days) mainly during the reproductive period. Our approach was able to accurately estimate plant N use in maize and to detect the dynamics of the in-season ^15^N fertilizer applications at development-stage-specific levels with a special focus on the reproductive period. This technique has high potential as an additional tool for phenotyping evaluation of genotypes with superior N use efficiency.

Although our emphasis was on applying ^15^N to maize plants during reproductive stages, this research observed a positive association of the allocation of ^15^Nu at late vegetative stage with the allocation of ^15^Nu during grain filling. Therefore, we propose that three N plant development stages (V15, R2, and early R5) are promising growth stages to evaluate potential hybrids with superior NUE. Future research studies using the multi-stage pulse labeling approach for phenotyping purposes with limited budgets should prioritize sampling a higher amount of plants (at least four) rather than increasing the rates of labeled N fertilizer beyond those used in our studies.

## Conclusions

This research describes and evaluates the effectiveness of intentional multi-stage ^15^N applications that we term as a “multi-stage pulse labeling” method of ^15^N application to late vegetative and reproductive-stage maize in field experiments. It is a potentially superior phenotyping technique to identify hybrids superior in NUE because of improved post-silking DM and N gains compared to hybrids of earlier decades. One of the major benefits of the ^15^N technique is that it precisely differentiates N allocation in plant organs of diverse genotypes under various N management situations, and then our modified approach demonstrates the extent to which plants are still actively taking up N during reproductive growth stages in response to new soil N supply. Even at the R5 stage when minimal new DM is being accumulated, maize plants were still taking up N and mainly allocating it to the kernels (~70%).

Our results suggest that plant N uptake is strongly driven by the sink strength and source availability. Leaves were the main sink component up to the kernel set period (R2) and, from R4 stage onward, more than 50% of the ^15^N absorbed accumulated in the ears. The moderate N rate (112N) increased absolute ^15^N uptake at all growth stages relative to the 0N treatment. Plants more stressed due to N deficiency (0N) partitioned more ^15^Nu to the reproductive organs. The primary allocation of the new ^15^Nu at both N rates was similar to stems and leaves during the late vegetative period, then higher to the leaf component at R1 (~42%) and R2 stages (~38%), and then increased to the ears at R4 (~55%) and kernels at R5 stages (~70%). However, lower soil N availability and, consequently, higher N stress in 2014 prompted higher partitioning of ^15^Np to ears already at the R2 stage.

Hence, the multi-stage pulse labeling technique proved to be a reliable and precise phenotyping approach that seed companies and universities can implement to investigate genotypes with particular traits targeted at improved maize N use efficiency.

## Author contributions

AOV conducted field experiments, laboratory measurements, and statistics and initial writing. TV conceived of and directed the project, the data interpretation, and its writing and editing, JC assisted with the writing, TC provided institutional support for the project via Dow AgroSciences (where he was a maize genetics research leader in the Trait Product Development Department at the time), and TF provided the Purdue Isotope Lab resources and technical assistance with the data output from the mass spectrometer analyses of isotopic plant tissue samples.

### Conflict of interest statement

The authors declare that the research was conducted in the absence of any commercial or financial relationships that could be construed as a potential conflict of interest.

## Supplementary material

### Supplementary ^15^N methodology procedures

More details on the modified “multi-stage pulse labeling” approach can be viewed in Supplementary Section A. Supplementary data of individual hybrid responses can be viewed in Supplementary Section B.

## Abbreviations

^15^N: Isotopic Nitrogen
^15^Np: Proportional allocation of ^15^N uptake in plant components
^15^Nrec: ^15^N fertilizer recovery
^15^Nu: ^15^N uptake per unit area
DM: Aboveground plant dry matter
GY: grain yield
Nc: Total Nitrogen concentration
^15^Nrem: Remobilized per unit area
^15^NremEF: ^15^N Remobilization efficiency
Nu: Total Nitrogen uptake per unit area
NUE: Nitrogen use efficiency
Post^15^N: Post silking ^15^N uptake per unit area.
