# Supplementary material for: Applicability of a “Multi-Stage Pulse Labeling” 15N Approach to Phenotype N Dynamics in Maize Plant Components during the Growing Season
Source: Front Plant Sci. 2017 Aug 4;8:1360. doi: 10.3389/fpls.2017.01360 (PMC5543178; doi:10.3389/fpls.2017.01360)
Supplement: Supplementary file 1 [file Table1.docx]

**Supplementary Section B**

**Table S1.** **Effects of overall sidedress N rate (0 and 112) on ^15^N uptake per unit area (^15^Nu, kg ha^-1^) at multiple development stages in 2013.**

| **GS** | **N rate**  **(kg ha^-1^)** | **LVS**  **^15^Nu**  **(kg ha^-1^)** | **STM**  **^15^Nu**  **(kg ha^-1^)** | **HSK**  **^15^Nu**  **(kg ha^-1^)** | **EAR**  **^15^Nu**  **(kg ha^-1^)** | **COB**  **^15^Nu**  **(kg ha^-1^)** | **KRN**  **^15^Nu**  **(kg ha^-1^)** | **TTL**  **^15^Nu**  **(kg ha^-1^)** |
| --- | --- | --- | --- | --- | --- | --- | --- | --- |
| **V14** | **0** | 0.44 | 0.35 b | ---- | ---- | ---- | ---- | 0.79 b |
|  | **112** | 0.49 | 0.47 a | ---- | ---- | ---- | ---- | 0.96 a |
| **V16** | **0** | 0.66 b | 0.39 b | 0.21 | ---- | ---- | ---- | 1.28 b |
|  | **112** | 0.71 a | 0.54 a | 0.21 | ---- | ---- | ---- | 1.46 a |
| **ER1** | **0** | 0.57 | 0.37 b | 0.19 | ---- | ---- | ---- | 1.13 |
|  | **112** | 0.61 | 0.53 a | 0.17 | ---- | ---- | ---- | 1.30 |
| **R1** | **0** | 0.39 b | 0.22 b | 0.11 | 0.29 a | ---- | ---- | 1.01 b |
|  | **112** | 0.46 a | 0.29 a | 0.11 | 0.24 b | ---- | ---- | 1.09 a |
| **R2** | **0** | 0.30 b | 0.15 b | 0.09 | 0.54 b | ---- | ---- | 0.86 b |
|  | **112** | 0.42 a | 0.27 a | 0.09 | 0.78 a | ---- | ---- | 1.07 a |
| **R4** | **0** | 0.21 b | 0.10 b | 0.02 b | 0.33 b | ---- | ---- | 0.79 b |
|  | **112** | 0.35 a | 0.14 a | 0.03 a | 0.52 a | ---- | ---- | 1.06 a |
| **R5** | **0** | 0.10 b | 0.07 b | 0.02 b | 0.41 b | 0.02 b | 0.39 b | 0.60 b |
|  | **112** | 0.15 a | 0.09 a | 0.02 a | 0.64 a | 0.03 a | 0.61 a | 0.90 a |

- GS growth stage; LVS Leaf; STM Stem; HSK Husk; Ear = cob + kernel (KRN); TTL= Total.
- Means with no letters are not significant different from each other, and means with different letters are significantly different at p<0.05 level T test (LSD) within a growth stage.

**Table S2.** **Effects of overall sidedress N rate (0 and 112) on ^15^N uptake per unit area (^15^Nu, kg ha^-1^) at multiple development stages in 2014.**

| **GS** | **N rate**  **(kg ha^-1^)** | **LVS**  **^15^Nu**  **(kg ha^-1^)** | **STM**  **^15^Nu**  **(kg ha^-1^)** | **HSK**  **^15^Nu**  **(kg ha^-1^)** | **EAR**  **^15^Nu**  **(kg ha^-1^)** | **COB**  **^15^Nu**  **(kg ha^-1^)** | **KRN**  **^15^Nu**  **(kg ha^-1^)** | **TTL**  **^15^Nu**  **(kg ha^-1^)** |
| --- | --- | --- | --- | --- | --- | --- | --- | --- |
| **V15** | **0** | 0.31 b | 0.26 b | ---- | ---- | ---- | ---- | 0.56 b |
|  | **112** | 0.46 a | 0.39 a | ---- | ---- | ---- | ---- | 0.87 a |
| **R1** | **0** | 0.16 b | 0.12 b | 0.07 b | 0.10 b | ---- | ---- | 0.44 b |
|  | **112** | 0.37 a | 0.23 a | 0.10 a | 0.19 a | ---- | ---- | 0.89 a |
| **R2** | **0** | 0.14 b | 0.09 b | 0.05 b | 0.23 b | ---- | ---- | 0.51 b |
|  | **112** | 0.32 a | 0.15 a | 0.07 a | 0.41 a | ---- | ---- | 0.94 a |
| **R4** | **0** | 0.10 b | 0.06 b | 0.01 b | 0.25 b | 0.02 b | 0.23 b | 0.42 b |
|  | **112** | 0.21 a | 0.09 a | 0.02 a | 0.47 a | 0.03 a | 0.45 a | 0.79 a |
| **R5** | **0** | 0.07 b | 0.05 b | 0.01 b | 0.26 b | 0.01 b | 0.24 b | 0.39 b |
|  | **112** | 0.16 a | 0.07 a | 0.02 a | 0.50 a | 0.02 a | 0.48 a | 0.75 a |
| **R1R6** | **0** | 0.06 b | 0.07 b | 0.04 | 0.79 b | 0.07 | 0.72 b | 0.96 b |
|  | **112** | 0.12 a | 0.10 a | 0.04 | 1.28 a | 0.07 | 1.20 a | 1.53 a |

- GS growth stage; LVS Leaf; STM Stem; HSK Husk; STV Stover (leaf + stem + husk + cob); Ear = cob + kernel (KRN).
- Means with no letters are not significant different from each other, and means with different letters are significantly different at p<0.05 level T test (LSD) within a growth stage.

**Table S3. Hybrid effects on ^15^N uptake and its associated parameters at multiple development stages in 2013.**

Parameters include ^15^N fertilizer recovery (^15^Nrec, kg kg^-1 15^N applied), ^15^N uptake per unit area (^15^Nu, kg ha^-1^)^15^, and proportional allocation of ^15^N uptake (^15^Np, kg kg^-1^) in plant components on average of two N rates (0 and 112 kg N ha^-1^).

| GS | Main Effect | ^15^Nrec  (kg kg^-1^) |  | LVS ^15^Nu  (kg ha^-1^) | STM ^15^Nu  (kg ha^-1^) | HSK ^15^Nu  (kg ha^-1^) | TTL ^15^Nu  (kg ha^-1^) | LVS ^15^Np  (%) | STM ^15^Np  (%) | HSK ^15^Np  (%) |
| --- | --- | --- | --- | --- | --- | --- | --- | --- | --- | --- |
| **V14** | **Hybrid** |  |  |  |  |  |  |  |  |  |
|  | HY-1 | 0.29 ab |  | 0.47 | 0.46 | ---- | 0.93 ab | 52 | 48 | ---- |
|  | NUE-2 | 0.24 c |  | 0.43 | 0.33 | ---- | 0.76 c | 57 | 43 | ---- |
|  | NUE-3 | 0.27 abc |  | 0.43 | 0.45 | ---- | 0.88 abc | 50 | 50 | ---- |
|  | HY-4 | 0.31 a |  | 0.53 | 0.47 | ---- | 1.00 a | 53 | 47 | ---- |
|  | OLD-5 | 0.27 bc |  | 0.47 | 0.38 | ---- | 0.85 bc | 56 | 44 | ---- |
| AOV | N Rate | ** |  | ns | ** | ---- | ** | ns | ns | ---- |
|  | Hybrid | * |  | ns | ns | ---- | * | ns | ns | ---- |
|  | NR * Hyb. | ns |  | ns | ns | ---- | ns | ns | ns | ---- |
| **V16** | **Hybrid** |  |  |  |  |  |  |  |  |  |
|  | HY-1 | 0.42 abc |  | 0.69 | 0.41 | 0.20 | 1.33 | 51 | 32 | 15 |
|  | NUE-2 | 0.41 c |  | 0.65 | 0.46 | 0.19 | 1.29 | 49 | 36 | 15 |
|  | NUE-3 | 0.42 bc |  | 0.64 | 0.45 | 0.21 | 1.33 | 48 | 36 | 15 |
|  | HY-4 | 0.46 ab |  | 0.71 | 0.49 | 0.22 | 1.46 | 48 | 34 | 15 |
|  | OLD-5 | 0.46 a |  | 0.75 | 0.50 | 0.22 | 1.47 | 50 | 35 | 15 |
| AOV | N Rate | * |  | * | ** | ns | ** | ns | ** | * |
|  | Hybrid | * |  | ns | ns | ns | ns | ns | ns | ns |
|  | NR * Hyb. | ns |  | ns | ns | ns | ns | ns | ns | ns |
| **ER1** | **Hybrid** |  |  |  |  |  |  |  |  |  |
|  | HY-1 | 0.40 |  | 0.61 | 0.46 | 0.17 | 1.24 | 47 | 36 | 14 |
|  | NUE-2 | 0.36 |  | 0.56 | 0.43 | 0.15 | 1.15 | 49 | 37 | 14 |
|  | NUE-3 | 0.37 |  | 0.53 | 0.42 | 0.18 | 1.13 | 46 | 36 | 15 |
|  | HY-4 | 0.42 |  | 0.60 | 0.45 | 0.20 | 1.25 | 45 | 34 | 15 |
|  | OLD-5 | 0.41 |  | 0.63 | 0.48 | 0.19 | 1.31 | 49 | 37 | 14 |
| AOV | N Rate | ns |  | ns | ** | ns | ns | * | ** | ** |
|  | Hybrid | ns |  | ns | ns | ns | ns | ns | ns | ns |
|  | NR * Hyb. | ns |  | ns | ns | ns | ns | * | ns | ns |

*Ns, not significant; * = p<0.05; ** = p<0.01 T test (LSD).*

- GS, growth stage; LVS, Leaf; STM Stem; HSK Husk; Ear= Cob + Kernel (KRN); TTL= Total.
- Growth stages represent means of two locations (ACRE and PPAC) with the exception of V14 stage (only PPAC location) and Early R1 (ER1) which was only tested at the ACRE location in 2013.

**Continued Table S3. Hybrid effects on ^15^N uptake and its associated parameters at multiple development stages in 2013.**

| GS | Main Effects | ^15^Nrec  (kg kg^-1^) |  | LVS ^15^Nu  (kg ha^-1^) | STM ^15^Nu  (kg ha^-1^) | HSK ^15^Nu  (kg ha^-1^) | EAR ^15^Nu  (kg ha^-1^) | KRN ^15^Nu  (kg ha^-1^) | TTL ^15^Nu  (kg ha^-1^) | LVS ^15^Np  (%) | STM ^15^Np  (%) | HSK ^15^Np  (%) | EAR ^15^Np  (%) | KRN ^15^Np  (%) |
| --- | --- | --- | --- | --- | --- | --- | --- | --- | --- | --- | --- | --- | --- | --- |
| **R1** | **Hybrid** |  |  |  |  |  |  |  |  |  |  |  |  |  |
|  | HY-1 | 0.33 bc |  | 0.42 | 0.25 | 0.10 bc | 0.28 a | ---- | 1.05 bc | 40 | 23 | 10 | 27 a | ---- |
|  | NUE-2 | 0.29 c |  | 0.38 | 0.23 | 0.09 c | 0.22 b | ---- | 0.93 c | 41 | 25 | 10 | 24 ab | ---- |
|  | NUE-3 | 0.34 ab |  | 0.44 | 0.27 | 0.10 abc | 0.23 a | ---- | 1.09 ab | 40 | 25 | 10 | 25 ab | ---- |
|  | HY-4 | 0.38 a |  | 0.47 | 0.27 | 0.12 a | 0.32 a | ---- | 1.19 a | 40 | 23 | 11 | 27 a | ---- |
|  | OLD-5 | 0.32 bc |  | 0.42 | 0.25 | 0.12 ab | 0.22 b | ---- | 1.01 bc | 42 | 25 | 12 | 21 b | ---- |
| AOV | N Rate | * |  | ** | ** | ns | ** | ---- | * | ** | ** | ns | ** | ---- |
|  | Hybrid | ** |  | ns | ns | * | ** | ---- | ** | ns | ns | ns | * | ---- |
|  | NR * Hyb. | ns |  | ns | ns | ns | ns | ---- | ns | ns | ns | ns | ns | ---- |
| **R2** | **Hybrid** |  |  |  |  |  |  |  |  |  |  |  |  |  |
|  | HY-1 | 0.30 |  | 0.33 b | 0.21 | 0.07 | 0.62 | ---- | 0.94 | 35 | 22 | 8 | 34 | ---- |
|  | NUE-2 | 0.28 |  | 0.33 b | 0.18 | 0.11 | 0.63 | ---- | 0.89 | 37 | 22 | 12 | 29 | ---- |
|  | NUE-3 | 0.31 |  | 0.39 a | 0.21 | 0.08 | 0.68 | ---- | 1.0 | 38 | 21 | 9 | 32 | ---- |
|  | HY-4 | 0.31 |  | 0.39 a | 0.20 | 0.09 | 0.68 | ---- | 0.99 | 39 | 21 | 9 | 31 | ---- |
|  | OLD-5 | 0.32 |  | 0.38 ab | 0.24 | 0.09 | 0.71 | ---- | 1.0 | 38 | 23 | 9 | 29 | ---- |
| AOV | N Rate | ** |  | ** | ** | ns | ** | ---- | ** | ** | ** | * | ** | ---- |
|  | Hybrid | ns |  | * | ns | ns | ns | ---- | ns | ns | ns | ns | ns | ---- |
|  | NR * Hyb. | ns |  | ns | ns | ns | ns | ---- | ns | ns | ns | ns | ns | ---- |
| **R4** | **Hybrid** |  |  |  |  |  |  |  |  |  |  |  |  |  |
|  | HY-1 | 0.28 |  | 0.27 | 0.11 | 0.02 | 0.40 | ---- | 0.87 | 30 | 13 | 3 b | 54 | ---- |
|  | NUE-2 | 0.28 |  | 0.28 | 0.12 | 0.03 | 0.43 | ---- | 0.90 | 30 | 14 | 3 b | 53 | ---- |
|  | NUE-3 | 0.29 |  | 0.28 | 0.12 | 0.03 | 0.43 | ---- | 0.91 | 30 | 13 | 4 a | 53 | ---- |
|  | HY-4 | 0.31 |  | 0.32 | 0.12 | 0.03 | 0.47 | ---- | 1.00 | 31 | 13 | 3 b | 54 | ---- |
|  | OLD-5 | 0.30 |  | 0.26 | 0.13 | 0.03 | 0.41 | ---- | 0.96 | 26 | 14 | 3 b | 57 | ---- |
| AOV | N Rate | ** |  | ** | ** | ** | ** | ---- | ** | ** | ns | ns | ** | ---- |
|  | Hybrid | ns |  | ns | ns | ns | ns | ---- | ns | ns | ns | ** | ns | ---- |
|  | NR * Hyb. | ns |  | ns | ns | ns | ns | ---- | ns | ns | ns | ns | ns | ---- |
| **R5** | **Hybrid** |  |  |  |  |  |  |  |  |  |  |  |  |  |
|  | HY-1 | 0.23 |  | 0.12 | 0.07 | 0.01 | 0.51 | 0.49 | 0.72 | 16 | 10 | 2 ab | 71 | 69 a |
|  | NUE-2 | 0.22 |  | 0.12 | 0.08 | 0.01 | 0.50 | 0.47 | 0.71 | 16 | 12 | 2 ab | 69 | 66 b |
|  | NUE-3 | 0.23 |  | 0.13 | 0.08 | 0.02 | 0.51 | 0.48 | 0.74 | 18 | 10 | 3 a | 69 | 65 b |
|  | HY-4 | 0.24 |  | 0.13 | 0.07 | 0.01 | 0.55 | 0.53 | 0.76 | 17 | 9 | 2 b | 72 | 70 a |
|  | OLD-5 | 0.24 |  | 0.12 | 0.08 | 0.01 | 0.54 | 0.52 | 0.75 | 16 | 11 | 2 ab | 72 | 69 a |
| AOV | NR | ** |  | ** | ** | ** | ** | ** | ** | ns | ns | ns | * | ** |
|  | Hybrid | ns |  | ns | ns | ns | ns | ns | ns | ns | ns | * | ns | ** |
|  | NR * Hyb. | ns |  | ns | ns | ns | ns | ns | ns | * | ns | ns | ns | ns |

**Table S4. Hybrid effects on ^15^N uptake and its associated parameters at multiple development stages in 2014.**

Parameters include ^15^N fertilizer recovery (^15^Nrec, kg kg^-1 15^N applied), ^15^N uptake per unit area (^15^Nu, kg ha^-1^)^15^, and proportional allocation of ^15^N uptake (^15^Np, kg kg^-1^) in plant components on average of two N rates (0 and 112 kg N ha^-1^).

| GS | Main Effect | ^15^N rec  (kg kg^-1^) |  | LVS ^15^Nu  (kg ha^-1^) | STM ^15^Nu  (kg ha^-1^) | HSK ^15^Nu  (kg ha^-1^) | EAR ^15^Nu  (kg ha^-1^) | KRN ^15^Nu  (kg ha^-1^ | TTL ^15^Nu  (kg ha^-1^) | LVS ^15^Np  (%) | STM ^15^Np  (%) | HSK ^15^Np  (%) | EAR ^15^Np  (%) | KRN ^15^Np  (%) |
| --- | --- | --- | --- | --- | --- | --- | --- | --- | --- | --- | --- | --- | --- | --- |
| V15 | **Hybrid** |  |  |  |  |  |  |  |  |  |  |  |  |  |
|  | HY-1 | 0.37 |  | 0.38 | 0.36 a | ---- | ---- | ---- | 0.77 | 49 c | 47 | ---- | ---- | ---- |
|  | NUE-2 | 0.30 |  | 0.37 | 0.26 b | ---- | ---- | ---- | 0.63 | 59 a | 41 | ---- | ---- | ---- |
|  | NUE-3 | 0.33 |  | 0.36 | 0.33 a | ---- | ---- | ---- | 0.70 | 51 bc | 47 | ---- | ---- | ---- |
|  | HY-4 | 0.36 |  | 0.41 | 0.35 a | ---- | ---- | ---- | 0.76 | 54 abc | 46 | ---- | ---- | ---- |
|  | OLD-5 | 0.34 |  | 0.40 | 0.30 ab | ---- | ---- | ---- | 0.72 | 56 ab | 42 | ---- | ---- | ---- |
| AOV | N Rate | ** |  | ** | ** | ---- | ---- | ---- | ** | ns | ns | ---- | ---- | ---- |
|  | Hybrid | ns |  | ns | * | ---- | ---- | ---- | ns | * | ns | ---- | ---- | ---- |
|  | NR * Hyb. | ns |  | ns | ns | ---- | ---- | ---- | ns | ns | ns | ---- | ---- | ---- |
| R1 | **Hybrid** |  |  |  |  |  |  |  |  |  |  |  |  |  |
|  | HY-1 | 0.33 |  | 0.26 | 0.18 | 0.08 | 0.18 a | ---- | 0.70 | 35 b | 26 | 13 | 26 a | ---- |
|  | NUE-2 | 0.31 |  | 0.25 | 0.19 | 0.08 | 0.13 b | ---- | 0.65 | 38 b | 30 | 13 | 19 cd | ---- |
|  | NUE-3 | 0.29 |  | 0.25 | 0.16 | 0.09 | 0.13 b | ---- | 0.62 | 39 b | 25 | 14 | 21 bc | ---- |
|  | HY-4 | 0.34 |  | 0.28 | 0.17 | 0.09 | 0.17 a | ---- | 0.71 | 39 b | 23 | 13 | 24 ab | ---- |
|  | OLD-5 | 0.31 |  | 0.29 | 0.17 | 0.07 | 0.11 b | ---- | 0.65 | 45 a | 25 | 13 | 17 d | ---- |
| AOV | NR | ** |  | ** | ** | ** | ** | ---- | ** | ** | ns | ** | ns | ---- |
|  | Hybrid | ns |  | ns | ns | ns | ** | ---- | ns | ** | ns | ns | ** | ---- |
|  | NR * Hyb. | ns |  | ns | ns | ns | ns | ---- | ns | ns | ns | ** | ns | ---- |
| R2 | **Hybrid** |  |  |  |  |  |  |  |  |  |  |  |  |  |
|  | HY-1 | 0.35 b |  | 0.21 | 0.11 c | 0.060 ab | 0.36 a | ---- | 0.74 b | 27 | 16 c | 8 b | 49 a | ---- |
|  | NUE-2 | 0.32 bc |  | 0.21 | 0.12 bc | 0.065 a | 0.29 b | ---- | 0.68 bc | 30 | 18 ab | 10 a | 41 bc | ---- |
|  | NUE-3 | 0.35 b |  | 0.25 | 0.14 ab | 0.068 a | 0.29 b | ---- | 0.74 b | 33 | 19 a | 10 a | 38 c | ---- |
|  | HY-4 | 0.42 a |  | 0.27 | 0.15 a | 0.063 a | 0.40 a | ---- | 0.88 a | 30 | 17 bc | 8 b | 45 ab | ---- |
|  | OLD-5 | 0.28 c |  | 0.20 | 0.09 d | 0.045 b | 0.26 b | ---- | 0.60 c | 32 | 15 c | 8 b | 45 ab | ---- |
| AOV | NR | ** |  | ** | ** | ** | ** | ---- | ** | ** | ** | ** | ns | ---- |
|  | Hybrid | ** |  | ns | ** | * | ** | ---- | ** | ns | ** | * | ** | ---- |
|  | NR * Hyb. | ns |  | ns | ns | ns | ns | ---- | ns | ns | ns | ns | ns | ---- |

**Continued Table S4.** **Hybrid effects on ^15^N uptake and its associated parameters at multiple development stages in 2014.**

| GS | Main Effect | ^15^N rec  (kg kg^-1^) |  | LVS ^15^Nu  (kg ha^-1^) | STM ^15^Nu  (kg ha^-1^) | HSK ^15^Nu  (kg ha^-1^) | EAR ^15^Nu  (kg ha^-1^) | KRN ^15^Nu  (kg ha^-1^ | TTL ^15^Nu  (kg ha^-1^) | LVS ^15^Np  (%) | STM ^15^Np  (%) | HSK ^15^Np  (%) | EAR ^15^Np  (%) | KRN ^15^Np  (%) |
| --- | --- | --- | --- | --- | --- | --- | --- | --- | --- | --- | --- | --- | --- | --- |
| R4 | **Hybrid** |  |  |  |  |  |  |  |  |  |  |  |  |  |
|  | HY-1 | 0.28 |  | 0.15 | 0.08 | 0.02 | 0.34 | 0.31 | 0.59 | 26 a | 14 a | 3 bc | 57 b | 53 b |
|  | NUE-2 | 0.27 |  | 0.15 | 0.07 | 0.02 | 0.35 | 0.32 | 0.58 | 25 a | 13 a | 3 bc | 59 b | 54 b |
|  | NUE-3 | 0.26 |  | 0.13 | 0.07 | 0.02 | 0.33 | 0.30 | 0.55 | 24 ab | 14 a | 3 ab | 60 b | 54 b |
|  | HY-4 | 0.32 |  | 0.18 | 0.09 | 0.02 | 0.39 | 0.37 | 0.67 | 26 a | 14 a | 2 c | 58 b | 55 b |
|  | OLD-5 | 0.30 |  | 0.15 | 0.06 | 0.02 | 0.40 | 0.38 | 0.63 | 23 b | 10 b | 4 a | 64 a | 61 a |
| AOV | NR | ** |  | ** | * | ** | ** | ** | ** | ** | ** | ns | ns | ns |
|  | Hybrid | ns |  | ns | ns | ns | ns | ns | ns | * | * | ** | ** | ** |
|  | NR * Hyb. | ns |  | ns | ns | ns | ns | ns | ns | ns | ns | ns | ns | ns |
| R5 | **Hybrid** |  |  |  |  |  |  |  |  |  |  |  |  |  |
|  | HY-1 | 0.25 |  | 0.12 b | 0.05 | 0.02 | 0.35 | 0.33 | 0.53 | 21a | 11 | 3 | 65 bc | 62 |
|  | NUE-2 | 0.29 |  | 0.12 b | 0.07 | 0.02 | 0.41 | 0.39 | 0.61 | 19b | 11 | 3 | 67 ab | 63 |
|  | NUE-3 | 0.26 |  | 0.10 b | 0.06 | 0.02 | 0.37 | 0.34 | 0.55 | 19b | 11 | 4 | 67abc | 62 |
|  | HY-4 | 0.29 |  | 0.14 a | 0.06 | 0.02 | 0.39 | 0.38 | 0.61 | 22a | 11 | 3 | 64 c | 62 |
|  | OLD-5 | 0.26 |  | 0.10 b | 0.05 | 0.02 | 0.38 | 0.37 | 0.55 | 19b | 10 | 3 | 69 a | 66 |
| AOV | NR | ** |  | ** | * | ** | ** | ** | ** | ** | ** | * | * | ** |
|  | Hybrid | ns |  | ** | ns | ns | ns | ns | ns | ** | ns | ns | * | ns |
|  | NR * Hyb. | ns |  | ns | * | ns | ns | ns | ns | * | ns | ns | ns | ns |
| R1R6 | **Hybrid** |  |  |  |  |  |  |  |  |  |  |  |  |  |
|  | HY-1 | 0.60 |  | 0.09 | 0.09 | 0.04 | 1.06 | 0.99 | 1.28 | 7 a | 7 | 3 | 83 | 77 |
|  | NUE-2 | 0.52 |  | 0.08 | 0.08 | 0.04 | 0.90 | 0.82 | 1.10 | 7 a | 7 | 4 | 82 | 75 |
|  | NUE-3 | 0.58 |  | 0.10 | 0.07 | 0.05 | 1.02 | 0.94 | 1.23 | 7 a | 6 | 4 | 83 | 76 |
|  | HY-4 | 0.62 |  | 0.11 | 0.09 | 0.04 | 1.07 | 1.02 | 1.31 | 8 a | 7 | 3 | 82 | 78 |
|  | OLD-5 | 0.62 |  | 0.08 | 0.08 | 0.05 | 1.11 | 1.05 | 1.32 | 6 b | 7 | 4 | 84 | 79 |
| AOV | NR | ** |  | ** | ** | ns | * | ** | ** | ** | ns | ** | ns | ** |
|  | Hybrid | ns |  | ns | ns | ns | ns | ns | ns | * | ns | ns | ns | ns |
|  | NR * Hyb. | ns |  | ns | ns | ns | ns | ns | ns | ns | ns | ns | ns | ns |

*Ns, not significant; * = p<0.05; ** = p<0.01 T test (LSD).*

- GS, growth stage; LVS, Leaf; STM Stem; HSK Husk; Ear= Cob + Kernel (KRN); TTL= Total.
- Growth Stages represent means of one location (ACRE) in 2014.


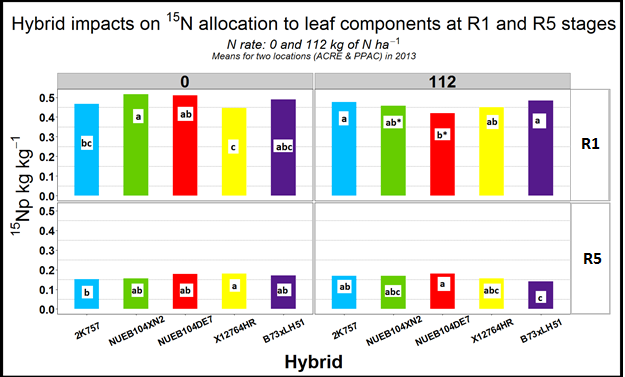
 **Figure S1.** Hybrid impacts on ^15^N allocation to leaf components (kg kg^-1^) at R1 and R5 stages. R1 is the “Early R1” stage for ACRE location only in 2013. R5 is the combined means for two locations (ACRE and PPAC) in 2013.
