# Supplementary material for: Applicability of a “Multi-Stage Pulse Labeling” 15N Approach to Phenotype N Dynamics in Maize Plant Components during the Growing Season
Source: Front Plant Sci. 2017 Aug 4;8:1360. doi: 10.3389/fpls.2017.01360 (PMC5543178; doi:10.3389/fpls.2017.01360)
Supplement: Supplementary file 2 [file DataSheet1.DOCX]

## **Supplementary Section A**

## **Methodology Procedures**

The detailed lab procedure can be reviewed in this section.

**Isotope Lab Analysis**

Plant materials were analyzed in the Purdue Stable Isotope (PSI) Laboratory located in the Department of Earth and Atmospheric Sciences at Purdue University. This PSI lab was under the supervision of Prof. Dr. Timothy Filley.

In this lab, meticulous cleaning was fundamental in order to avoid ^15^N cross contamination among samples. Using gloves, samples were prepared in ascending order from the smallest to the highest anticipated concentration of ^15^N in plant components. Every tool was repeatedly washed and rinsed at least three times, with soap and water, distilled water, followed by the solvent ethanol (Pure 190 proof, 95%), then dried before the preparation of the next sample. Distilled water was used to remove the ^15^N from the instruments and working surface, followed by ethanol, to eliminate the remaining grease from plant tissues in the instruments. The countertops were covered with aluminum foil which facilitated cleaning, and this foil was replaced after every plant component.

For the subsampling process, 25% of the whole sample (coarse ground material) was transferred to glass vials with caps (28 x 57 mm 4 dram). This coarsely ground tissue was ground to a fine powder with a stainless steel mixer mill (Restch, Haan, Germany) for 60 seconds. Prior to mass spectrometry analysis, samples were weighed and placed on tin capsules. Weights varied per plant component and development stage from 1.5 to 4.0 µg in 2013 and from 2.5 – 8.0 µg in 2014. Folded sample tins were placed into a plastic tray, labeled and identified with each weight recorded manually to correspond to the sample analysis in the mass spectrometer.

Total N concentration (Nc) and delta ^15^N (δ^15^N) were determined by combustion of the plant material in a Carlo Erba EA1108 C/N Elemental Analyzer (EA) (Sercon Ltd, Crewe, UK) coupled to 20 - 22 Stable Isotope-ratio Continuous Flow Mass Spectrometer (IRMS) calibrated for measuring N.

**Mass Spectrometry N Analysis**

In order to acquire an effective and precise isotopic evaluation it is crucial to avoid ^15^N cross-contamination during the sample preparation. To ensure there was no ^15^N residue in the chopping or coarse grinding process, a cross contamination test was developed at the Agronomy Department using unlabeled plants harvested at the R4 and R5 stages. Unlabeled samples were ground before and after grinding a labeled material. As per the usual procedure, the plant sample grinder was cleaned with a paint brush to remove residues remaining in the blades and vacuumed for removal of the small particles. The unlabeled intermediate-stage samples were analyzed in the Isotope Lab in the mass spec and confirmed the absence of ^15^N carryover in the chopping or coarse grinding process.

In the Isotope Laboratory all instruments utilized for labeled analysis were carefully adjusted and calibrated. The inside combustion tubes and quartz liners in the Carlo Erba EA 1108 Elemental Analyzer were always verified prior to sample examination due to the possible mineral deposition from previous analysis. In the flash combustion procedure, the total N in plant tissues was converted to gas molecules N_2_ through a sequential combustion (1050°C) and reduction (680 °C). In this process a tunable electron impact ion source and a magnetic sector mass filter were used to separate and quantify the relative proportion of ^14^N^14^N (with atomic mass equal to 28), ^14^N ^15^N (29) and ^15^N^15^N (30). Alteration in atomic masses of the N elements enabled the ions estimation by their mass ratio. The isotopic ratio of the reference standards (NIST 1547 Peach Leaf) were used to calculate the ^15^N abundance in the plant samples. Calibration lines were built for each run in the mass spec through the regression analysis of the beam area with its relative sample weight (µg) to finally provide total N concentration (Nc) and the proportional amount of ^15^N and ^14^N were determined for each individual sample. If for any technical reason a value fell outside the calibration range, the samples were reweighed and reanalyzed.

The continuous flow mass spectrometry enabled evaluation of several highly enriched samples at a time (50) including blanks and standards, evaluating ~15 samples per hour. Samples were dropped individually into loading trays and completely isolated from each other in the combustion tubes. Throughout each run in the IRMS blank samples were interspersed after every three labeled samples to insure no ^15^N carry over. Delta^15^N represented the proportional amount of isotopic N in parts per mil (δ^15^N ‰) in the sample relative to an international isotope standard of Air (0.3663 %) (Shearer and Kohl 1986). To further improve the precision of analysis in the IRMS, three reference standards were used in the isotope lab; peach tree leaves (NIST1547) containing 2.84 ^14^N + 1.36 δ^15^N‰ units relative to N_2,_ USGS41 1.15 ^14^N + 47.57 δ^15^N‰, and an in-house reference standard consisting of ^15^N - enriched maple tree leaves with 1.73 ^14^N + 162 δ^15^N‰.

Comparisons between identical samples with different weights, 4 or 8 µg, proved that heavier weights facilitated the detection of the ^15^N in plant components with low Nc while maintaining consistent total Nc values in the samples. For this reason, sample weights varied somewhat per plant component and development stage. Heavier samples were necessary for plant components containing lower Nc especially at late - season sampling times. For example, with stems, husks and cobs (~ 0.35% Nc) at late season, samples sizes were up to 4 µg in 2013 and 8 µg in 2014. Because of the considerable amount of N present in the leaves (~ 0.8 to 3% Nc) and kernels (~ 1% Nc) throughout the season, leaf and kernel sample weights were held consistent across years. Leaf weight samples increased from 1.5 to 3 µg as the season progressed and kernels were weighed to about 2.5 µg. In 2013 plants received a higher dose of ^15^N fertilizer than in 2014 (3.2 to 2.1 kg of ^15^N ha^-1^, respectively), which required larger sample weights for the low N components (stem, husk and cob) in 2014.

Several authors have confirmed the estimation of isotope ratio to accurately quantify ^15^N abundance rather than the direct evaluation of absolute isotope values (Hoefs 2009; Fry 2006). Hayes 2002 stated that long-term isotopic instability may affect absolute values, but that the isotope ratio remains constant. The latter verifies the isotope ratio approach (IR=^15^N/^14^N) as an accurate method to estimate total N concentration (Nc) and ^15^N abundance (δ^15^N) in mass spectrometry analysis. Therefore, the mass spectrometry analysis proved to be a reliable method to quantify labeled or non-labeled N in plant tissues able to capture very low Nc amounts in plant materials.

## References

**Fry B.** 2006. Stable isotopes in ecology. Springer, *New York. Ecology* **88,** 802.

**Hayes JM.** 1982. Fractionation et al.: An introduction to isotopic measurements and terminology. *Spectra* **8,** 3-8.

**Hoefs J.** 2009. Stable isotope geochemistry. *Mercury* **46**. Doi:10.1180/minmag. 1982.046.338.35.

**Shearer G, Kohl DH.** 1986. N_2_-Fixation in Field Settings: Estimations Based on Natural ^15^N Abundance. *Australian Journal of Plant Physiology* **13,** 699–756. Doi:10.1071/PP9860699c.
